# Gut–immune–PVAT axis is involved in ethanol-induced abdominal aortic dysfunction via IL-17RA, TLR4, and FPR1 signaling

**DOI:** 10.1080/19490976.2026.2734648

**Published:** 2026-09-21

**Authors:** Carla Brigagão Pacheco da Silva, Vanessa Fernandes Rodrigues, Daniela Carlos, Maria Cecília Jordani, Leandra Naira Zambelli Ramalho, José Teles de Oliveira-Neto, Jefferson Elias-Oliveira, Marília Cristina Oliveira Souza, Rafael Menezes da Costa, Mirele Resende Machado, Milene Tavares Fontes, Paul Townsend Jr, Rinaldo Rodrigues dos Passos, Ricardo Bernardino de Paula, Tiago Januário Costa, Cintia Vieira dos Santos, Gabriel Henrique Savietto, Daniel Rodrigues, Christiane Becari, Cameron G McCarthy, R. Clinton Webb, Camilla Ferreira Wenceslau, Rita C Tostes

**Affiliations:** a Department of Pharmacology, Ribeirao Preto Medical School, University of São Paulo, Ribeirão Preto, SP, Brazil; b Department of Cell Biology and Anatomy, University of South Carolina School of Medicine, Cardiovascular Translational Research Center, Columbia, SC, United States; c Ribeirão Preto Medical School, Department of Biochemistry and Immunology, University of São Paulo, Ribeirão Preto, SP, Brazil; d Department of Surgery and Anatomy, Ribeirão Preto Medical School, University of São Paulo, Ribeirão Preto, SP, Brazil; e Department of Pathology, Ribeirão Preto Medical School, University of São Paulo, Ribeirão Preto, SP, Brazil; f Department of Biomolecular Sciences, School of Pharmaceutical Sciences of Ribeirão Preto, University of São Paulo, Ribeirão Preto, SP, Brazil; g Institute of Health Sciences, Federal University of Jatai, Jatai, GO, Brazil; h Department of Pharmacology, Institute of Biomedical Science, University of São Paulo, São Paulo, São Paulo, SP, Brazil; i Department of Biological Sciences, School of Dentistry of Bauru, University of São Paulo, Bauru, Bauru, São Paulo, SP, Brazil

**Keywords:** Alcohol exposure, gut microbiota, perivascular adipose tissue, fMLP, LPS, Th17, IL-17A.

## Abstract

Alcohol consumption and gut dysbiosis are risk factors for cardiovascular diseases (CVD), yet their mechanistic interplay remains unclear. We investigated whether gut microbiota-associated signaling modulates ethanol-induced abdominal aorta and perivascular adipose tissue (PVAT) dysfunction. Male C57BL/6J mice subjected to a chronic plus binge ethanol protocol exhibited leaky gut, systemic inflammation, and vascular dysfunction, along with greater systemic exposure to microbiota-derived products (fMLP and LPS) and immune response activation, including M1 macrophage polarization and Th17 expansion. Ethanol-induced vascular hyporesponsiveness was linked to FPR1 upregulation, while smooth muscle FPR1 conditional deletion prevented this effect. PVAT-intact aorta from ethanol-treated mice exhibited increased contractile responses compared with PVAT-devoid vessels, indicating PVAT dysfunction. This phenotype was attenuated by pharmacological inhibition or genetic deletion of FPR1. Notably, these PVAT-dependent alterations were absent in germ-free, Rag1^-/-^, IL-17RA^-/-^, or TLR4^-/-^ mice, supporting a role for gut microbiota-driven immune response signaling. Redox imbalance also contributed to vascular and PVAT dysfunction. Our findings demonstrate that ethanol exposure triggers a gut–redox cascade which elicits the Th17/TLR4 axis and targets the PVAT to drive vascular injury. In conclusion, positioning PVAT as a central integrator of gut microbiota-derived signals identifies the gut–vascular interface as a promising therapeutic target for alcohol-related CVD.

## Graphical abstract

**Figure:**
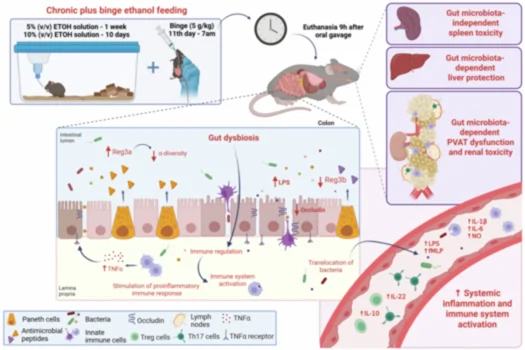

Gut microbiota-dependent and independent signaling in multi-organ toxicity induced by chronic plus binge ethanol consumption in male mice. Created in BioRender.

## 1. Introduction

According to the Global Burden of Diseases, Injuries, and Risk Factors Study (GBD) 2019, alcohol use represents the leading risk factor for disability-adjusted life-years (DALYs) in the 25–49-year age group.^1^ In the same year, 2.44 million deaths were attributed to ethanol consumption, with over 84% occurring among men.^1^ Furthermore, approximately 780,000 deaths each year are associated with ethanol-attributable cardiovascular diseases (CVD).^2^ The relationship between ethanol consumption and CVD risk depends on both the volume of intake and drinking patterns.^2^ A recent study has suggested that any level of ethanol intake is related to increased risk for hypertension in men, while consumption of 1 to 2 drinks per day was not associated with increased risk for women.^3^ For best health outcomes in hypertensive patients, a current clinical guidelines from the American Heart Association recommend complete abstinence from alcohol.^4^ Despite these associations, the mechanisms linking ethanol exposure to CVD remain incompletely defined.

The gut microbiota has emerged as a potential contributor to ethanol-induced cardiovascular system dysfunction, acting directly through metabolites and peptides or indirectly via modulation of the immune system.^5^ Our recent work demonstrated that ethanol intake modifies the mouse gut microbiome in a drinking pattern- and sex-dependent manner, which may influence susceptibility to ethanol-related pathologies.^6^ Specifically, male mice subjected to a chronic plus binge ethanol protocol exhibited pronounced gut dysbiosis, characterized by reduced bacteria diversity and richness, alongside other microbial alterations.^6^ Given that this drinking pattern has been linked to cardiovascular dysfunction,^7^ ^,^ ^8^ these findings suggest a potential mechanistic role for the microbiota in this process.

Pro-oxidative and pro-inflammatory molecules, such as hydrogen peroxide (H_2_O_2_) and trimethylamine *N*-oxide (TMAO), are associated with gut dysbiosis-related vascular dysfunction.^9^ N-formyl-methionyl-leucyl-phenylalanine (fMLP), a bacterial-derived peptide, and lipopolysaccharide (LPS), a glycolipid component of the bacterial outer membrane, act as synergistic inducers of inflammation, a process involving Toll-like receptor 4 (TLR4) signaling.^10^ In this context, TLR4 also contributes to ethanol-induced gut dysbiosis and the inflammatory response.^11^

The fMLP peptide activates formyl peptide receptors (FPRs), G protein-coupled receptors (GPCRs) expressed in various cells and tissues, including phagocytic leukocytes, endothelium, spleen, liver, mesenteric resistance arteries, and the heart.^12^ In the cardiovascular system, fMLP binds to FPR1 and modulates vascular tone.^12^ However, the specific effects of FPR1 are controversial, as they appear to depend on the species and vascular bed.^12-14^ For example, FPR1-induced hypercontractility has been observed in mesenteric resistance arteries from Dahl salt-sensitive rats fed a low-salt diet,^13^ whereas fMLP-induced FPR1 activation has been associated with vasorelaxation in mesenteric resistance arteries from Wistar rats.^12^ While a protective role of FPR2 against ethanol-induced liver damage has been established,^15^ the contribution of FPR1 to the effects of ethanol remains unexplored.

Accumulating evidence points to a role for dysfunctional perivascular adipose tissue (PVAT) in mediating ethanol‑associated vascular injury.^16-18^ PVAT is a metabolically active organ composed of adipocytes, fibroblasts, immune cells, mast cells, stem cells, and nerves.^16^ ^,^ ^19^ ^,^ ^20^ Under healthy conditions, PVAT contributes to vascular homeostasis through the release of vasorelaxing factors, such as nitric oxide (NO), H_2_O_2_, angiotensin-1 to 7, hydrogen sulfide (H_2_S), adiponectin, and prostacyclin. In contrast, under pathophysiological conditions, PVAT undergoes functional remodeling toward a pro-contractile and pro-inflammatory phenotype. This state is characterized by increased production of reactive oxygen species (ROS), angiotensin II, noradrenaline, alongside reduced NO bioavailability.^16^ ^,^ ^21^ Inflammation within PVAT has emerged as a potential mediator of gut dysbiosis-induced metabolic and cardiovascular dysfunction.^22^ However, the mechanisms linking microbiota-derived signals to PVAT dysfunction and vascular impairment during ethanol exposure remain unclear.

The immune system plays a significant role in the pathophysiology of ethanol-induced disease.^23^ Notably, increased interleukin (IL)-17A expression in T cells has been described in male mice subjected to chronic plus binge ethanol consumption.^24^ Furthermore, PVAT influences T cell differentiation and activation, promoting inflammation that can precede the onset of hypertension.^25^ The development of memory T cells in PVAT has also been linked to hypertension progression.^26^ This is particularly relevant given that abdominal PVAT exhibits a more pro-inflammatory phenotype than thoracic PVAT.^27^ Although IL-17 has been broadly associated with inflammation and vascular dysfunction,^26^ ^,^ ^28-30^ the specific role of Th17/IL-17A/IL-17RA signaling in regulating vascular tone and PVAT function during ethanol exposure remains poorly defined.

Therefore, the present study aimed to investigate whether gut microbiota-dependent signaling pathways contribute to vascular and PVAT dysfunction induced by chronic plus binge ethanol consumption. We hypothesized that fMLP/FPR1, TLR4, and Th17/IL-17RA signaling modulates abdominal aortic vascular tone and PVAT remodeling during ethanol exposure through mechanisms involving oxidative stress and inflammation. Importantly, germ-free (GF) mice were used as a mechanistic loss-of-function system at the host–microbiota interface and not as a standalone associative model. Using complementary genetic deletion models and pharmacological approaches, we further sought to define the mechanistic contribution of the gut microbiota in linking ethanol-induced gut dysbiosis to abdominal aortic vascular impairment and PVAT dysfunction.

## 2. Materials and methods

### 2.1. Animal studies

All animal procedures were approved by the Institutional Animal Care and Use Committees of the Ribeirao Preto Medical School (University of Sao Paulo, Brazil; CEUA #1007/2021) and the University of South Carolina School of Medicine (USA; IACUC #2648-101791-060723). All experiments were conducted in accordance with the National Institutes of Health Guide for the Care and Use of Laboratory Animals and ARRIVE guidelines, including formal randomization procedures and predefined exclusion criteria.

Eight-week-old male mice were used for all experiments. The following strains were included: C57BL/6J wild-type (WT), C57BL/6NTac germ-free (GF), IL-17 receptor subunit A knockout (IL-17RA^-/-^), recombination-activating gene-1 knockout (Rag1^-/-^), TLR4 knockout (TLR4^-/-^), and smooth muscle cell (SMC)-specific FPR1-deficient mice (FPR1^fl/fl^/Myh11-CreER^T2^; referred to as FPR1^-/-^ throughout the text) (Supplementary Figure S1; Supplementary Tables S1-S2). The inclusion of GF and genetically modified strains targeting innate and adaptive immune pathways was designed to dissect microbiota-dependent and immune-mediated mechanisms underlying ethanol-induced vascular dysfunction. Additional methodological details are provided in the Supplementary Material and Methods. All animals were housed under controlled environmental conditions (12-hour light/dark cycle; temperature- and humidity-controlled rooms) with *ad libitum* access to standard laboratory chow and water or ethanol solution, where applicable.

The exclusive use of male mice was based on robust prior evidence demonstrating that chronic plus binge ethanol exposure induces a markedly greater disruption of gut microbiota in males than females.^6^ Moreover, chronic plus binge ethanol exposure does not elicit measurable abdominal aortic dysfunction in female WT mice under identical conditions (Supplementary Figure S2), thereby limiting mechanistic interpretability. Accordingly, the study design was optimized to maximize biological signal and mechanistic resolution in a microbiota-dependent context.

Animals were monitored daily, and predefined humane endpoints were applied. Criteria for removal from the study included body weight loss exceeding 15%, impaired physical appearance (e.g., sunken eyes, body condition score ≤2 on a 5-point scale), abnormal behavior (e.g., lethargy or reduced responsiveness), failure to gain weight during growth, or other signs of compromised health status.

### 2.2. Experimental design

Mice were treated according to a modified National Institute on Alcohol Abuse and Alcoholism (NIAAA) model of chronic plus binge ethanol consumption, originally described by Bertola et al. (2013)^31^ and adapted by our group.^6^ This protocol combines chronic ethanol exposure with an acute binge challenge and is widely used in experimental studies of alcohol-induced tissue injury.^8^ ^,^ ^32-37^ Our group has previously reported blood alcohol concentrations (BACs) of approximately 300 mg/dL (~0.3% w/v) in male mice using this protocol.^6^ These levels are comparable to those observed in individuals with severe alcohol use disorder,^38^ consistent with high-risk drinking patterns.

Animals were randomly assigned to either a control group or a chronic plus binge ethanol group. The detailed experimental design has been previously described.^6^ Briefly, the ethanol exposure protocol consisted of a one-week adaptation period with *ad libitum* access to a 5% (v/v) ethanol solution, followed by 10 d of *ad libitum* access to a 10% (v/v) ethanol solution. On day 11, a single binge dose of ethanol (5 g/kg) was administered via oral gavage. Control animals received water throughout the experimental period. Both control and ethanol-treated mice were fasted for 9 h after receiving oral gavage (water for controls, ethanol for the ethanol-treated group). They were then anesthetized with isoflurane (5% in 100% O_2_) prior to euthanasia via exsanguination via cardiac puncture. Blood and tissue samples were collected, immediately processed, or stored at −80 °C until further analysis.

### 2.3. Assessment of organ alterations

Following euthanasia, the heart, spleen, kidneys, and liver were carefully removed from control and ethanol-treated mice. Organ wet weights were recorded and normalized to body weight (g/g). In addition, the colon and cecum were removed, and their lengths were measured and normalized to body weight (cm/g).

### 2.4. Assessment of intestinal permeability

Intestinal permeability was assessed by measuring circulating levels of fluorescein isothiocyanate (FITC)-dextran and zonulin. After a 12-hour fast, mice were administered FITC-dextran (400 mg/kg body weight; Sigma-Aldrich) via oral gavage. Animals with evidence of incomplete gavage were excluded from the analysis. Blood samples were collected 4 hours after administration and centrifuged at 10,000 × g for 2 minutes. Serum FITC-dextran levels were quantified by fluorescence using excitation and emission wavelengths of 485 nm and 535 nm, respectively, and standard curves were used for quantification. Results are expressed as μg/mL.

For zonulin quantification, blood samples were collected into EDTA-containing tubes and centrifuged at 3,000 × g for 10 minutes at 4 °C. Plasma zonulin levels were determined using a commercially available Mouse Zonulin ELISA kit (#MBS2603528, MyBioSource, San Diego, CA, USA), following the manufacturer's instructions. Results are expressed as ng/ml.

### 2.5. Assessment of colonic gene expression

Total RNA was extracted from mouse colon samples using TRIzol reagent (Invitrogen). The quantity and purity of RNA were determined using a NanoDrop spectrophotometer (NanoDrop Technologies, Wilmington, DE, USA), with an acceptable 260/280 nm ratio for nucleic acid purity set at ≥1.8. Subsequently, 2 μg of total RNA was reverse transcribed into complementary DNA (cDNA) using the High-Capacity cDNA Reverse Transcription kit (Life Technologies®) according to the manufacturer's protocol. Quantitative real-time PCR (RT-qPCR) was performed on a StepOne Real-Time PCR System (Applied Biosystems®), using SYBR™ Green PCR Master Mix (#4309155, Applied Biosystems®). Samples that failed to amplify or exhibited non-specific melting curves were excluded from the analysis. Relative gene expression was calculated using the comparative 2^−ΔΔCt^ method, with normalization to the housekeeping gene *β*-actin. Primer sequences are listed in Supplementary Table S3.

### 2.6. Assessment of bacterial translocation

Bacterial translocation was assessed by quantifying colony-forming units (CFU) in blood and circulating endotoxin (lipopolysaccharide, LPS) levels. Whole blood was collected aseptically via cardiac puncture and immediately processed for CFU quantification. Aliquots (50 μL) of blood were spread onto brain-heart infusion agar plates using a sterile loop and incubated at 37 °C for 48 hours. Bacterial colonies were then counted and expressed as CFU/mL of blood. The rate of negative blood cultures (no growth under the conditions used) was recorded as an internal control for contamination.

Serum LPS levels, used as a marker of intestinal barrier disruption and bacterial translocation, were determined using a quantitative chromogenic Limulus Amebocyte Lysate (LAL) assay (Pyrochrome kit, #C1500-5, Associates of Cape Cod Incorporated, East Falmouth, MA), following the manufacturer’s instructions. Samples were heat-inactivated (70 °C for 10 minutes) to reduce potential enzymatic interference and analyzed in duplicate. To control for assay interference, endotoxin-free materials were used throughout, and each sample was also tested with an endotoxin spike recovery control (spiked with 0.1 EU/mL standard) and a blank matrix control. Samples with spike recovery below 50% or above 200% were excluded. Results are expressed as endotoxin units (EU)/mL.

### 2.7. Assessment of bacterial presence in PVAT

To evaluate bacterial presence and alterations in microbial composition, abdominal PVAT samples were collected under sterile conditions and stored at −80 °C until analysis. DNA extraction was performed using a commercial kit (DNeasy® Blood & Tissue Kit, Qiagen), following the instructions. Bacterial translocation to PVAT was assessed by 16S gene expression. For qPCR analysis of 16S DNA expression, 10 ng of DNA and 5 µM of 16S primers were used (forward: 5′-AACAGGATTAGATACCCTGGTAG-3′; reverse: 5′-GGTTCTTCGCGTTGCATC-3′).

Relative abundance of bacteria from the Bacillota (synonym Firmicutes), Bacteroidota (synonym Bacteroidetes), Pseudomonadota (synonym Proteobacteria), Actinomycetota (synonym Actinobacteria), and Tenericutes phyla was determined using primers targeting the 16S rRNA gene for total bacteria (Eubacteria) and phylum-specific primers (listed in Supplementary Table S3). The differences (ΔCt) between the cycle threshold (Ct) values of Eubacteria and specific bacterial phylum (Ct_phylum_ − Ct_Eubacteria_) were used to obtain normalized levels of each bacterial group (2^−ΔCt^).^39^ The relative abundance of each bacterial phylum was obtained after normalization to the control groups. For heatmap visualization, data were scaled relatively to the control group, and deviations in ethanol-treated mice were represented using a color gradient. Results should be interpreted as semi-quantitative estimates of bacterial abundance.

### 2.8. LC-MS/MS Determination of plasma fMLP Levels

Blood samples were collected into heparin-treated tubes and centrifuged for 15 minutes at 10,000 × g at 4 °C, to obtain the plasma, and stored at −80 °C. A stock solution of fMLP (1 mg/mL, ab141806, Abcam) was prepared in methanol (Sigma-Aldrich®, USA). Working solutions and serial dilutions were prepared in a methanol: water mixture (30:70, v/v). Ultrapure water (18.2 MΩ·cm) was obtained from a Milli-Q system (Millipore®, USA). Samples were analyzed using an HPLC system (Shimadzu®, Japan) coupled to a triple quadrupole mass spectrometer (Quattro-LC, Micromass®, UK) with electrospray ionization (ESI). Data acquisition was performed using MassLynx 4.0 software. The MS/MS method was performed in positive ESI mode, with the following settings: capillary voltage, 4,000 V; cone voltage, 20 V; extractor voltage, 2 V; and desolvation temperature, 250 °C. The monitored transitions were m/z 439 > 273 (10 eV), 439 > 166 (14 eV), and 439 > 420 (10 eV). Chromatographic separation was performed using a Pinnacle DB AQ C18 column (100 × 2.1 mm, 5 µm, Restek®) at 0.3 mL/minute in isocratic mode with 30% acetonitrile and 70% water (0.1% formic acid). Retention times were 3.33 minutes (fMLP) and 4.17 minutes (ormetoprim—internal standard). Plasma samples (300 µL) were diluted with phosphate buffer (200 µL, pH 7.0) and spiked with 10 µL of internal standard. Samples were extracted and purified employing solid-phase extraction (SPE) using Oasis HLB PRIME cartridges (Waters®) and eluted with 1.0 mL methanol. After evaporation, the samples were reconstituted in 50 μL of methanol and 150 μL of water. Calibration curve and quality control samples were performed using blank human plasma, due to limited availability of analyte-free murine plasma, spiked with fMLP (1-50 ng/mL), which was processed as described above.

### 2.9. Determination of serum TMAO levels

Blood samples were collected and kept at room temperature for 15–20 minutes. Subsequently, the samples were centrifuged at 3,000 × g for 20 minutes to obtain serum, which was stored in a freezer at −80 °C until analysis. Serum TMAO concentrations were determined following the instructions of the commercial Mouse Trimethylamine-*N*-Oxide ELISA Kit (TMAO BT-LAB Kit, #EA0116Mo, Bioassay Technology Laboratory, Shanghai, China) and expressed in µmol/L.

### 2.10. Assessment of vascular function

Suprarenal segments of abdominal aorta (AA), 2 mm in length, with intact endothelium, and in the presence or absence of PVAT [AA-PVAT (+) and AA-PVAT (–), respectively], were mounted on DMT pin myographs (Danish MyoTech, Aarhus, Denmark) and maintained at 37 °C Krebs buffer (130 mmol/L NaCl; 4.7 mmol/L KCl; 1.18 mmol/L H_2_PO_4_; 1.17 mmol/L MgSO_4_.7H_2_O; 14.9 mmol/L NaHCO_3_; 0.026 mmol/L Na_2_EDTA; 1.6 mmol/L CaCl_2_.2H_2_O; and 5.5 mmol/L glucose) with 5% CO_2_ and 95% O_2_. Vessels were equilibrated under a passive tension of 4.5 mN. Vascular smooth muscle viability was assessed by contracting vessels with 90 mmol/L KCl. However, contractile responses to pharmacological agonizts were not normalized to KCl-induced contraction due to significant differences in KCl responsiveness between AA-PVAT (+) and AA-PVAT (–) segments (data not shown). Such normalization could introduce bias in the interpretation of PVAT-mediated effects; therefore, all responses are presented as absolute tension (mN), allowing direct and physiologically relevant comparisons between groups. Endothelial integrity was evaluated by pre-contracting vessels with phenylephrine (PE; 1 µmol/L) and subsequently stimulating them with acetylcholine (ACh; 10 µmol/L) to elicit endothelium-dependent vasodilation. Rings that failed to achieve at least 70% relaxation were excluded. Cumulative concentration–response curves to PE (0.1 nmol/L to 100 µmol/L) were performed. Endothelium-dependent and -independent relaxation were evaluated using cumulative additions of ACh and sodium nitroprusside (SNP), respectively, after pre-contraction of AA segments with PE (0.1 µmol/L). E_max_ (maximum response), p*D*_2_ [negative logarithm of the molar concentration of the agonist that promoted 50% of the maximum response (-log EC_50_), indicating potency], and AUC (area under the curve) were calculated from concentration–response curves, which were fitted using a non-linear interactive fitting program (GraphPad Prism 10.5.0).

To investigate the mechanisms underlying ethanol-induced AA and PVAT dysfunction, concentration–response curves to PE were performed in segments incubated for 30 minutes with the following pharmacological agents: fMLP (FPR1 agonist, 10 µmol/L, #ab141806, Abcam); cyclosporin H (CsH, a FPR1 antagonist, 10 µmol/L, #sc-20301A, Santa Cruz Biothecnology), mitoquinone (MitoQ, a mitochondria-targeted antioxidant, 100 nmol/L, #ab285406, Abcam); Nω-Nitro-L-arginine methyl ester (L-NAME, a non-selective inhibitor of the enzyme nitric oxide synthase (NOS), 100 µmol/L, #N5751, Sigma-Aldrich); 1400 W (specific iNOS inhibitor, 1 µmol/L, #W4262, Sigma-Aldrich); or Tiron [superoxide anion (O_2_^•−^) scavenger, 100 µmol/L, #sc-253699, Santa Cruz Biotechnology]. The concentrations employed were based on previous studies.^12^ ^,^ ^40^ ^,^ ^41^

Pharmacological agents were dissolved in water or dimethyl sulfoxide (DMSO). Final DMSO concentrations were maintained below 0.5%, a level that does not affect basal vascular tone or agonist-mediated contraction or relaxation.^42^

### 2.11. Histopathology evaluation of AA and colon

Samples of AA and colon were isolated for histopathological analysis. Suprarenal segments of AA were dissected, fixed in 4% paraformaldehyde (PFA) for 24 hours, and then embedded in paraffin. For histological analysis, AA sections (7 μm thick) were stained with hematoxylin and eosin (H&E). Collagen accumulation in AA was assessed using picrosirius red staining. For the colon, distal segments were isolated, washed with cold PBS to remove luminal contents, and fixed in 10% PFA, followed by paraffin embedding. Colon sections (5 µm thick) were stained with H&E. The degree of colonic inflammation was scored from 0 to 3 based on established criteria from previous studies.^43^ ^,^ ^44^

### 2.12. Tail cuff measurements of blood pressure

Systolic blood pressure (SBP) was determined in conscious mice by a noninvasive blood pressure system (CODA® High Throughput System, Torrington, USA). Animals were placed in acrylic cylinders while the tail remained exposed. Mice were acclimatized for 3 d before SBP measurement. Mice were heated at 36–37 °C for 5 minutes, and then measurements were performed with 3 acclimatization readings, and 10 regular readings were performed in one cycle. Blood pressure was measured in the tail of the mice using the Volume Pressure Recording (VPR) sensor technology and an occlusion cuff kit attached to the proximal tail portion of the mice. The mean of these 10 measurements was considered the SBP.

### 2.13. Evaluation of mechanical properties

Suprarenal segments of the AA (~2 mm in length) were isolated and subjected to mechanical analysis using a tissue puller system (DMT 560TP, Danish Myo Technology). Vessels were mounted and stretched at a constant rate of 20 μm/s until rupture. Stress-strain curves were generated as previously described.^45^ Stress was calculated as force per unit cross-sectional area (F/A), where F represents the applied force and A corresponds to the vessel cross-sectional area. Strain was defined as the relative change in length (∆L/L_0_), where L_0_ represents the initial length of the vessel segment. The AUC was calculated using GraphPad Prism (version 10.5.0) as an index of overall mechanical behavior.

### 2.14. Measurement of pro-inflammatory cytokines

Cytokine levels were quantified in plasma, PVAT and colon samples. Blood was collected via cardiac puncture, using heparinized syringes and centrifuged (10,000 × g, 15 minutes, 4 °C) to obtain plasma. For tissue cytokine analysis, PVAT and colon samples were homogenized in ice-cold phosphate-buffered saline (PBS, pH 7.4) and centrifuged (10,000 × g, 15 minutes, 4 °C) to collect the supernatant. Cytokine concentrations were measured using enzyme-linked immunosorbent assay (ELISA) kits, following the manufacturer’s instructions. The following kits were used: TNF-*α*, IL-6, IL-10, IL-17 (DuoSet - ELISA Development System DY410, DY406, DY417, and DY421, respectively; R&D Systems, Minneapolis, MN, USA), IL-1β, and IL-22 (ELISA MAX Deluxe Set, Biolegend, CA, USA; #432604 and #436304, respectively). Cytokine levels in PVAT and colon homogenates were normalized to the total protein concentration of each sample and are expressed as pg/mg of protein. Plasma cytokine levels are expressed as pg/mL.

### 2.15. Quantification of ROS and NO Levels

Fresh segments of AA with intact PVAT were embedded in Tissue-Tek® - OCT™ (Sakura Finetek, Torrance, CA, USA), rapidly frozen in liquid nitrogen, and stored at −80 °C until sectioning. The sections (7 µm thick) were mounted on slides, incubated (30 minutes, 37 °C) with dihydroethidium (DHE, 1 μmol/L) diluted in PBS (pH 7.4), and washed three times with PBS. ROS were quantified *in situ* from images captured (at 400x magnification) using a fluorescence microscope (Carl Zeiss Microscopy Ltd., Germany). Tissue sections were visualized at an excitation and emission wavelengths of 550 nm and 570 nm, respectively. For each animal, the fluorescence intensity was measured in 4 separate sections, and an arithmetic mean was calculated per slide. Quantification was performed using ImageJ software (NIH, Bethesda, MD, USA). Results are expressed as fluorescence intensity in arbitrary units.

Hydrogen peroxide (H_2_O_2_) concentrations in the AA and PVAT were measured using the Amplex® Red Hydrogen Peroxide/Peroxidase Assay Kit (#A22188, Invitrogen, Carlsbad, USA). Briefly, tissues were homogenized in ice-cold assay buffer (50 mM KH_2_PO_4_, 1 mM EGTA, and 150 mM sucrose, pH 7.4), and centrifuged (10,000 × g, 5 minutes, 4 °C). Aliquots (50 µL) of the resulting supernatant were then incubated with the Amplex Red reagent. H₂O₂ generation was assessed fluorometrically, using excitation and emission wavelengths of 530 and 590 nm, respectively. Fluorescence values were normalized to the total protein content of each sample, and results are expressed as nmol/mg protein.

To assess mitochondrial ROS generation, O_2_^•-^ and H_2_O_2_ levels were determined in the AA by lucigenin chemiluminescence and Amplex Red assays, respectively. Vessels were isolated and incubated with (2-(2,2,6,6-Tetramethylpiperidin-1-oxyl-4-ylamino)-2-oxoethyl)triphenylphosphonium chloride (MitoTEMPO; 100 nmol/L; #SML0737, Sigma-Aldrich), a mitochondria-targeted antioxidant, at 37 °C for 1 hour. Following the incubation, samples were frozen and maintained at −80 °C until analysis. The chemiluminescent probe lucigenin (bis-*N*-methylacridinium nitrate) was used to determine ROS generation, specifically O_2_^•-^, as previously described,^17^ and results were expressed as relative light units (RLU)/mg of protein. The Amplex Red assay was performed as described above.

NO levels were measured in plasma, AA, and PVAT using a Sievers NO Analyzer (Sievers 280i NOA, Sievers, Boulder, CO, USA) as previously described.^46^ In this method, nitrate and nitrite (NO*x*) were reduced to NO, which was subsequently detected by ozone-based chemiluminescence. Samples were deproteinized and analyzed under standardized conditions to minimize variability. Results are expressed as µM for plasma and µM/mg protein for tissue samples.

### 2.16. Mitochondrial respiration measurements

Mitochondrial respiration was determined in a high-resolution respirometry—Oroboros Oygraph 2k-FluoRespirometer (Oroboros Instruments, Innsbruck, Austria) –, as previously described.^47^ Briefly, oxygen consumption was determined in permeabilized AA (2-6 mg wet weight) incubated with MiR05 buffer (0.5 mM EGTA, 3 mM MgCl_2_, 60 mM potassium lactobionate, 20 mM taurine, 10 mM KH_2_PO_4_, 20 mM HEPES, 110 mM sucrose, and 0.1% (w/v) BSA, pH 7.1) at 37 °C. Mitochondrial function was assessed using a substrate-uncoupler-inhibitor titration (SUIT) protocol with the following compounds: a) malate, pyruvate, glutamate (MPG): substrates for inducing proton leak; b) ADP (adenosine diphosphate): used to measure oxidative phosphorylation capacity through Complex I; c) succinate (S): used to measure combined oxidative phosphorylation through complex I + II; d) cytochrome C (Cyt C): to assess the integrity of the mitochondrial outer membrane; e) CCCP (carbonyl cyanide 3-chlorophenylhydrazone, a mitochondrial uncoupler): to measure maximum capacity of the ETS (electron transfer system); f) rotenone (Rot, a CI inhibitor): to measure ETS capacity supported by Complex II alone; g) antimycin A (Ama, a CIII inhibitor): to measure ROX (residual oxygen consumption), representing non-mitochondrial respiration; h) Ascorbate + TMPD (N-N-N′-N′-Tetramethyl-p-phenylenediamine dihydrochloride): to determine CIV (cytochrome c oxidase) activity; i) sodium Azide (Azd, a CIV inhibitor).^48^ All assays were carried out in a hyperoxygenated environment to prevent oxygen diffusion limitations. The oxygen consumption rate (OCR) was measured in picomoles of O_2_ per second (pmol·s^−1^) and then normalized to the wet weight of the AA sample (expressed in pmol.s^−1^.mg wet wt^−1^).^47^

### 2.17. Flow cytometry analysis of neutrophils, macrophages, and lymphocytes

Abdominal aortic PVAT and cecal lymph nodes were collected for immune cell profiling. A cell suspension from PVAT was prepared as described in a previous study.^16^ The cecal lymph nodes were isolated and processed for flow cytometric analysis according to a published protocol.^44^ Following isolation and digestion, the total cell counts for both PVAT and cecal lymph nodes were determined. Cells were then resuspended in PBS containing 1% BSA. For each experiment, a total of 1 × 10^6^ cells were used. Following incubation with Fc block^TM^ (1:300, BioRad) (30 minutes at 4 °C) to block nonspecific binding. Cells were stained with FVS-BV605 (#565694, BD Biosciences) to assess viability and incubated with the following fluorochrome-conjugated antibodies (1:200): CD11b-APC-Cy7 (#557657, BD Biosciences), Ly6g-APC (#560599, BD Biosciences), F4/80-PerCP (#123126, Biolegend), CD11c-FITC (#553801, BD Biosciences), TLR2-BV421 (#565908, BD Biosciences), CD45-PeCy7 (#202225, Biolegend), CD206-PE (#141706, Biolegend), CD3-Pe (#553064, BD Biosciences), and CD4-FITC (#553729, BD Biosciences) to external labeling. After cell permeabilization with the Transcription Factor Staining Buffer Set (Invitrogen), transcription factors were stained using the following antibodies (1:100): RORɣt-PerCP-efluor710 (#46-6988-82, Invitrogen), FoxP3-APC (#17-5773, eBioscience), and Tbet-BV421 (#563318, BD Biosciences). Labeled cells were acquired using a FACSCanto II Cell Analyzer (BD Biosciences), and data were analyzed using the FlowJo™ Software (BD Biosciences, Franklin Lakes, NJ, USA). Gating strategies are shown in Supplementary Figure S3.

### 2.18. Immunofluorescence assay

Suprarenal segments of the AA were embedded in OCT, and cryosections (7 µm thick) were fixed in 2% paraformaldehyde for 10 minutes at room temperature. After washing with PBS, the sections were blocked and permeabilized with PBS (0.01 M) supplemented with 1% BSA and 0.5% Triton X-100 for 2 hours at 4 ºC. Primary antibodies were applied overnight at 4 °C: rabbit anti-FPR1 (1:100, #FPR1-101AP, FabGennix International) and Alexa Fluor™ 488–conjugated monoclonal anti-*α*-smooth muscle actin (1:100, #53-9760-82, Invitrogen). After washing, sections were incubated with Alexa Fluor™ 680–conjugated anti-rabbit IgG (1:200, #AF680D) for 90 minutes at room temperature, counterstained with DAPI, and mounted with Fluoromount-G™. Confocal images were obtained using a Leica Stellaris 5 microscope (20 × objective, dichroic detection). Acquisition parameters (laser power and gain) were kept constant across groups to allow direct comparisons. Quantification of FPR1 expression was performed from two sections per animal and five randomly selected fields per section.

### 2.19. Western immunoblotting

Protein expression of FPR1 was determined in the PVAT from WT and GF mice, using a western immunoblotting assay. Briefly, adipose tissue was homogenized in lysis buffer (cOmplete Lysis-M, #04719956001, Roche, Germany) containing phosphatase inhibitors (PhosSTOP EASYpack, #04906845001; cOmplete^TM^ ULTRA Tablets, Mini, EASYpack, #05892970001, Roche, Germany) and sonicated. Equivalent amounts of protein (30 µg) were separated by sodium dodecyl sulfate-polyacrylamide gel electrophoresis (SDS-PAGE), followed by transfer to nitrocellulose membranes. Membranes were blocked with 5% dried milk (Blotting Grade Blocker #1706404XTU, BioRad) in TBS-T for 1 hour at room temperature and incubated with primary antibody anti-FPR1 (1:500, #FPR1-101AP, FabGenni) overnight at 4 °C. After washing with TBS-T, membranes were incubated with secondary antibody anti-rabbit (1:2500, #711-035-152, Jackson ImmunoResearch) for 90 minutes at room temperature. Protein bands were visualized using a luminescent solution (ECL). To account for potential variations in protein loading, membranes were stained with Red Ponceau, and normalization was performed using the total protein signal, rather than housekeeping proteins. Densitometric analysis of western blotting images was conducted using ImageJ (National Institutes of Health).

### 2.20. Protein-protein interaction network construction

A protein-protein interaction (PPI) network was generated using the Search Tool for the Retrieval of Interacting Genes/Proteins (STRING) database (version 12.0; https://string-db.org/). STRING integrates known and predicted protein associations derived from curated databases, experimental evidence, and computational predictions, encompassing both physical and functional interactions.^49^ This analysis was conducted as an exploratory, hypothesis-generating approach to provide integrative, pathway-level context for the identified targets. A medium confidence interaction score threshold of 0.4 was applied for network construction.

### 2.21. Statistical analysis

Data are presented as mean ± standard error of the mean (SEM), with sample sizes (*n*) indicated in the respective figures and tables. Statistical analyzes were performed using GraphPad Prism® (version 10.5.0, GraphPad Software Inc., San Diego, CA, USA). Samples were excluded from the final analysis based on pre-established technical criteria, including visible hemolysis, failure to meet quality control thresholds (e.g., RT-qPCR amplification failure), or accidental injury during oral gavage (e.g., FITC-dextran assay). Statistical outliers were identified using the ROUT method (Q = 1%) and excluded where applicable. Data normality was assessed using the Shapiro-Wilk test (*α* = 0.05). For normally distributed data, statistical comparisons were performed using paired or unpaired Student’s *t*-tests, one-way ANOVA, or two-way ANOVA, followed by Bonferroni’s post hoc test or uncorrected Fisher’s least significant difference (LSD) test for multiple comparisons, as appropriate. For non-normally distributed data, the Mann-Whitney test or Kruskal-Wallis test followed by Dunn’s post hoc test were employed. Statistical significance was defined as *p* < 0.05. Detailed statistical analysis information is provided in the Supplementary Material.

## 3. Results

### 3.1. Chronic plus binge ethanol exposure is associated with intestinal inflammatory responses and barrier-related alterations in the large intestine

We previously demonstrated that ethanol-induced alterations in gut microbiota composition depend on pattern of consumption and sex.^6^ To investigate whether the pronounced gut dysbiosis induced by chronic plus binge ethanol consumption in male mice is associated with intestinal alterations, we evaluated inflammatory markers, antimicrobial peptides, and intestinal permeability.

Histopathological analysis revealed no differences in colonic inflammation scores among groups (Supplementary Figure S4A). However, colonic levels of the proinflammatory cytokine TNF-*α* were significantly increased in the chronic plus binge ethanol group, whereas no changes were observed in the chronic or binge groups (Supplementary Figure S4B). Levels of IL-6, IL-1β, IL-17A, IL-22, and IL-10 were unchanged (Supplementary Figure S4C–G), indicating a modest inflammatory response at the molecular level that was not reflected in overt histopathological changes.

Given the role of regenerating islet-derived protein (Reg) family antimicrobial peptides in controlling bacterial load and modulating intestinal immune responses,^5^ we assessed their gene expression in the colon. *Reg3a* gene expression was significantly increased in all ethanol-treated groups, with higher levels in the chronic plus binge group (Supplementary Figure S4H). In contrast, *Reg3b* gene expression was significantly reduced in the chronic and chronic plus binge groups (Supplementary Figure S4I), whereas *Reg3g* gene expression remained unchanged (Supplementary Figure S4J).

In addition to antimicrobial peptides, tight junction proteins play a critical role in maintaining intestinal barrier integrity.^50^ Colonic *occludin* gene expression was significantly reduced in the binge and chronic plus binge groups (Supplementary Figure S4K). *Claudin-2* expression was also decreased in all ethanol-treated groups; however, its levels were higher in the binge group compared with the chronic and chronic plus binge groups (Supplementary Figure S4L). No changes were observed in *claudin-3* or *zonula occludens-1* (ZO-1) gene expression (Supplementary Figure S4M-N).

Consistent with these molecular alterations, intestinal permeability, assessed by FITC-dextran, was significantly increased in the binge and chronic plus binge groups (Supplementary Figure S5A). Altogether, these findings demonstrate that intestinal barrier-associated parameters are differentially regulated depending on the pattern of ethanol exposure. Additionally, given that claudin-2 is typically associated with increased intestinal permeability,^51^ its reduction contrasts with the overall barrier impairment observed, suggesting a complex tight junction remodeling.

### 3.2. Chronic plus binge ethanol exposure promotes systemic exposure to bacterial products and an inflammatory profile

We next investigated whether intestinal alterations were associated with bacterial translocation and systemic inflammation. Bacterial translocation into the circulatory system, assessed by CFU counts, was detected exclusively in the chronic plus binge group (Supplementary Figure S5B). This was accompanied by increased serum LPS levels (Table 1), consistent with enhanced systemic exposure to bacterial-derived components and intestinal barrier disruption. Elevated LPS levels were also observed in the chronic ethanol group but not following a single binge exposure (data not shown).

**Table 1.** Inflammatory markers in male WT mice.

Samples	Inflammatory markers	Control	Chronic plus binge ethanol
Serum (EU/mL)	LPS	0.022 ± 0.003 (8)	0.144 ± 0.057 (8)^a^
Abdominal aorta (pg/mg protein)	TNF-α	5069 ± 584 (8)	3108 ± 498 (7)^a^
	IL-1β	5590 ± 589 (8)	3349 ± 260 (8)^a^
	IL-6	1250 ± 172 (8)	854 ± 135 (8)^a^
	IL-17A	3124 ± 447 (5)	1296 ± 234 (5)^a^
	IL-22	978 ± 164 (5)	497 ± 75 (5)^a^
PVAT(pg/mg protein)	TNF-α	1436 ± 390 (4)	2216 ± 294 (4)
	IL-1β	2486 ± 258 (8)	7141 ± 1731 (8)^a^
	IL-6	1377 ± 351 (5)	1535 ± 317 (5)
	IL-17A	2068 ± 613 (5)	4283 ± 958 (5)^a^
	IL-22	216 ± 19 (4)	607 ± 165 (5)^a^

Data are presented as mean ± SEM. Samples sizes (*n*) for each experimental set are indicated in the table. Statistical analyzes were performed using Mann-Whitney test (for LPS) or unpaired Student’s *t* test (for cytokines).​​​​​

^a^ Versus the control group (*p* < 0.05). Abbreviations: IL, interleukin; LPS, lipopolysaccharide; PVAT: perivascular adipose tissue; TNF-*α*, tumor necrosis factor-alpha.

This phenotype was associated with systemic inflammatory profile in the chronic plus binge group, as evidenced by increased circulating levels of IL-1β, IL-6, IL-22, and NO (Supplementary Figure S5C–F), whereas TNF-*α*, IL-17A, and IL-10 levels remained unchanged (Supplementary Figure S5G–I). No significant alterations were observed in the chronic or binge groups (Supplementary Figure S5B–I).

Collectively, these findings indicate that chronic plus binge ethanol exposure is associated with a distinct systemic inflammatory phenotype, which was further investigated in subsequent experiments.

### 3.3. Chronic plus binge ethanol exposure induces organs alterations and impact AA vascular tone and PVAT function

We next evaluated whether systemic alterations were associated with changes in organ and tissue parameters. No differences were observed in colon or cecum length, nor in liver or heart weights (Supplementary Figure S6A–E). In contrast, size and weight of spleen and kidney were significantly reduced following chronic plus binge ethanol exposure (Supplementary Figure S6F–G).

**Figure 1.**
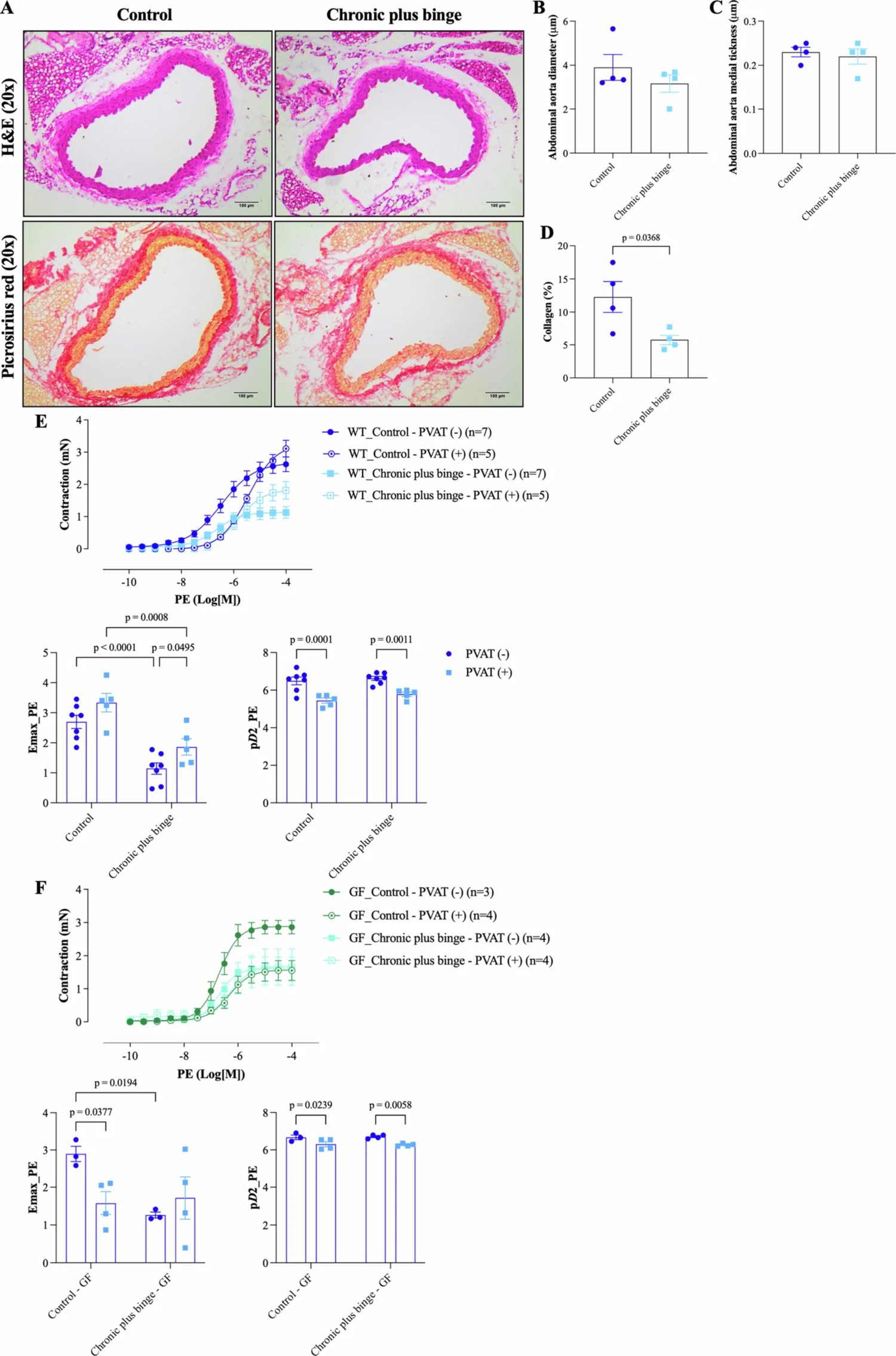
Absence of gut microbiota attenuates abdominal PVAT dysfunction but not aortic hyporesponsiveness induced by chronic plus binge ethanol exposure. (A) Representative H&E- (top) and Picrosirius Red-stained (bottom) cross-sections of the AA from WT control and ethanol-treated mice (scale bar, 100 μm; one representative animal per group). (B-C) Histomorphometric analysis of medial thickness (B) and aortic diameter (C). (D) Collagen content in the AA. (A–D) Analyzes were performed from the same set of samples (*n* = 4/group) from one independent experimental cohort. (E-F) Concentration–response curves to PE in AA segments with or without PVAT [PVAT (+) or PVAT (–), respectively] from WT (*n* = 5-7/group) (E) and GF (*n* = 3-4/group) (F) mice in control and ethanol groups. Upper, representative curves. Lower, summary data showing E_max_ and p*D*_2_, respectively. Vascular reactivity data were obtained from multiple experimental sessions (typically two animals per session) to achieve the final sample size under consistent experimental conditions. Sample sizes vary due to technical limitations of vascular reactivity assays and the limited availability of GF mice. Data are presented as mean ± SEM. Statistical analyzes were performed using the Mann-Whitney test (B), unpaired Student’s *t*-test (C-D), or two-way ANOVA followed by uncorrected Fisher’s LSD test for multiple comparisons (E-F) (*p* < 0.05; exact *p* values are indicated). Abbreviations: AA, abdominal aorta; E_max_, maximum response; GF, germ-free; H&E, hematoxylin and eosin; p*D*_2_, -log EC_50_; PE, phenylephrine; PVAT, perivascular adipose tissue; WT, wild-type.

In the cardiovascular system, no differences were observed in SBP (Supplementary Figure S7A) or AA morphology (Figure 1A–C). However, chronic plus binge ethanol exposure significantly reduced collagen content in the AA (Figure 1A, D), accompanied by a rightward shift in the stress–strain curve (Supplementary Figure S7B).

To assess AA vascular function, concentration–response curves were generated. Preliminary analyzes revealed regional heterogeneity in vascular reactivity, with infrarenal segments exhibiting greater contractile responses to PE than suprarenal segments (Supplementary Figure S7C). To avoid regional bias, subsequent experiments were performed using suprarenal segments. Ethanol exposure did not affect endothelium-dependent or -independent relaxation responses to ACh and NPS, respectively, indicating preserved relaxation capacity (Supplementary Figure S7D-E). In contrast, contractile responses to PE were significantly reduced in ethanol-treated mice, indicating vascular hyporesponsiveness (Figure 1E).

Given that PVAT is a key regulator of vascular homeostasis by modulating vascular tone and local inflammatory responses,^52^ PVAT function was also evaluated. Consistent with previous reports,^53^ PVAT did not exert an anti-contractile effect under basal conditions in control mice (Figure 1E). However, ethanol exposure altered abdominal PVAT function. In ethanol-treated mice, the presence of PVAT increased contractile responses compared with PVAT-denuded vessels, despite an overall reduction in vascular contractility compared with controls (Figure 1E). These data suggested that ethanol exposure may reprogram PVAT from a neutral to a pro-contractile modulator of vascular tone.

### 3.4. Gut microbiota differentially modulates PVAT responses to ethanol exposure

To assess the contribution of the gut microbiota, parallel experiments were performed in GF mice. No differences were observed in colon or cecum length, or heart weight between GF groups (Supplementary Figure S8A–D), whereas spleen weight was reduced in ethanol-treated GF mice (Supplementary Figure 8E). In contrast to WT mice (Supplementary Figure S6), ethanol consumption did not alter kidney weight in GF mice (Supplementary Figure S8F), while liver weight was increased (Supplementary Figure S8G).

Zonulin, a marker associated with intestinal permeability,^54^ was differentially modulated by ethanol. Plasma zonulin levels were significantly reduced in ethanol-treated WT mice, while in GF mice, ethanol exposure did not significantly alter zonulin levels compared with GF controls, despite a downward trend (Supplementary Figure S9A), suggesting a partially gut microbiota-dependent modulation of this response.

Functionally, ethanol also induced vascular hyporesponsiveness in AA-PVAT (–) segments of GF mice (Figure 1F). However, PVAT responses differed between groups. In GF control mice, abdominal PVAT showed a robust anti-contractile phenotype (Figure 1E-F). Notably, in ethanol-treated GF mice, contractile responses in AA-PVAT (+) segments were preserved and overlapped with those observed in GF controls (Figure 1F), indicating that ethanol failed to disrupt PVAT function in the absence of gut microbiota.

Emerging evidence suggests that bacterial infiltration is linked to altered PVAT function.^55^ Thus, we next evaluated bacterial presence within the PVAT. Ethanol exposure was associated with increased bacterial DNA content in PVAT from WT mice (Supplementary Figure S9B), accompanied by shifts in microbial composition, including increased relative abundance of Bacillota, Actinomycetota, and Tenericutes, and reduced Pseudomonadota (Supplementary Figure S9C). No significant alterations were observed in Bacteroidota (Supplementary Figure S9C).

### 3.5. Chronic plus binge ethanol exposure increases gut microbiota-derived fMLP levels

**Figure 2.**
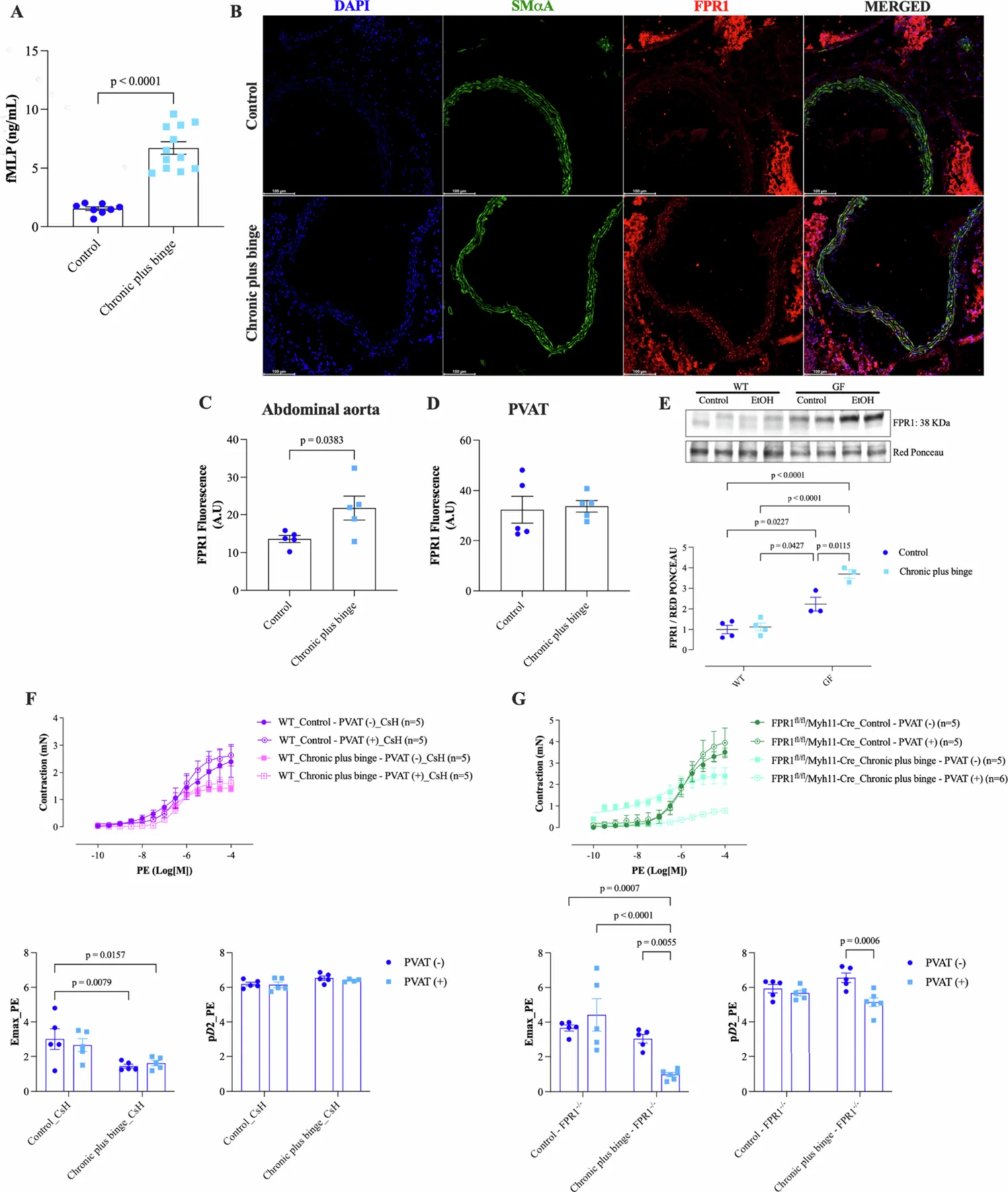
fMLP/FPR1 signaling contributes to AA and PVAT dysfunction following chronic plus binge ethanol exposure. (A) Plasma fMLP levels measured by LC-MS/MS (*n* = 8-12/group) in WT control and ethanol-treated mice. (B) Representative immunofluorescence images for DAPI (nuclei), SMαA (VSMCs) and FPR1 (scale bar, 100 μm) in WT control (top) and ethanol-treated (bottom) mice (one representative animal per group). (C-D) Quantification of FPR1 fluorescence in the AA (C) and PVAT (D) (*n* = 5/group). (E) FPR1 protein expression in PVAT from WT (*n* = 4/group) and GF (*n* = 3/group) mice assessed by immunoblotting. Full blots are provided in Supplementary Figure S10A-B. Data were obtained from one (B–D) or four (A, E) independent experimental cohorts. (F–G) Concentration–response curves to PE in AA segments with or without PVAT [PVAT (+) or PVAT (–), respectively] from WT mice in the presence of the FPR1 antagonist CsH (10 µmol/L; *n* = 5/group) (F), or in vessels from FPR1-deficient mice (*n* = 5-6/group) (G). Upper, representative curves. Lower, summary data showing E_max_ and p*D*_2_, respectively. Vascular reactivity data were obtained from multiple experimental sessions (typically two animals per session) to achieve the final sample size under consistent experimental conditions. Sample sizes vary due to technical limitations or limited availability of GF mice. Data are presented as mean ± SEM. Statistical analyzes were performed using unpaired Student’s *t*-test (A–D), two-way ANOVA followed by Bonferroni’s post hoc test (E), or two-way ANOVA followed by uncorrected Fisher’s LSD test for multiple comparisons (F, G) (*p* < 0.05; exact *p* values are indicated). Abbreviations: AA, abdominal aorta; CsH: cyclosporin H; E_max_, maximum response; EtOH, chronic plus binge group; fMLP, *N*-formyl–methionyl–leucyl–phenylalanine; FPR1, formyl peptide receptor 1; FPR1^-/-^, FPR1^fl/fl^/Myh11-CreER^T2^; GF, germ-free; LC-MS/MS, Liquid Chromatography with tandem mass spectrometry; p*D*_2_, -log EC_50_; PE, phenylephrine; PVAT, perivascular adipose tissue; VSMCs, vascular smooth muscle cells; WT, wild-type.

A recent study demonstrated that ethanol-induced gut microbial reorganization and subsequent increases in gut microbial metabolites contribute to CVD.^7^ Among these, TMAO has been associated with vascular and PVAT dysfunction,^22^ and the bacteria-derived peptide fMLP has been implicated in the modulation of vascular contractility.^12^ To determine whether these metabolites link the gut microbiota to ethanol-induced vascular dysfunction, circulating levels of TMAO and fMLP were quantified in WT mice. No differences in serum TMAO concentrations were observed between the groups (Supplementary Figure S9D). In contrast, plasma fMLP levels were significantly increased in ethanol-treated mice compared with controls (Figure 2A).

### 3.6. fMLP/FPR1 signaling modulates AA vascular tone and PVAT function

Given the increase in circulating fMLP (Figure 2A), and its role as a gut microbiota-derived agonist of FPR1, we investigated whether fMLP/FPR1 signaling is involved in ethanol-induced alterations. SMC-specific deletion of FPR1 prevented the ethanol-induced reduction in plasma zonulin levels (Supplementary Figure S9A), suggesting that FPR1 signaling may influence systemic responses associated with intestinal barrier regulation.

At the vascular level, chronic plus binge ethanol exposure significantly increased FPR1 protein expression in the AA (Figure 2B-C), whereas no changes were observed in PVAT from WT mice (Figure 2B, D-E; Supplementary Figure S10A-B). In contrast, FPR1 expression was upregulated in abdominal PVAT from ethanol-treated GF mice (Figure 2E; Supplementary Figure S10A-B), suggesting that gut microbiota status differentially regulates FPR1 expression in a tissue-specific manner. In addition, PVAT from GF mice exhibited higher basal FPR1 expression compared with WT controls (Figure 2E; Supplementary Figure S10A-B).

To determine the functional relevance of FPR1 signaling, both pharmacological inhibition and SMC-specific genetic deletion approaches were employed. Pharmacological inhibition of FPR1 with CsH did not reverse ethanol-induced vascular hyporesponsiveness in AA-PVAT (–) segments (Figure 2F), whereas SMC-specific deletion of FPR1 prevented this effect (Figure 2G). In contrast, CsH attenuated PVAT dysfunction in ethanol-treated mice (Figure 2F). Notably, ethanol exposure induced an anti-contractile PVAT phenotype in FPR1^-/-^ mice (Figure 2G).

We next investigated whether FPR1 signaling modulates PVAT function under basal conditions. Consistent with WT mice, PVAT from control FPR1^-/-^ mice did not exhibit an anti-contractile phenotype (Figure 2G). In WT control mice, pharmacological inhibition of FPR1 did not affect PVAT-mediated regulation of AA tone (Figure 2F; Supplementary Figure S10C), whereas activation with fMLP significantly reduced contractile responses in AA-PVAT (+) segments (Supplementary Figure S10C).

Collectively, these findings suggested the fMLP/FPR1 axis as a gut microbiota-sensitive signaling pathway that differentially regulates vascular tone and PVAT function. These results establish FPR1 as a key integrator of gut-derived microbial signals and vascular responses, revealing a compartment-specific mechanism linking microbiota-derived metabolites to ethanol-induced vascular dysfunction.

### 3.7. Chronic plus binge ethanol exposure alters immune cell profiles and cytokine release

In addition to bacteria-derived metabolites, immunological pathways may impact the gut–vascular axis, contributing to onset and progression of CVD.^5^ ^,^ ^9^ Therefore, we investigated whether chronic plus binge ethanol consumption modulates innate and adaptive immune responses.

**Figure 3.**
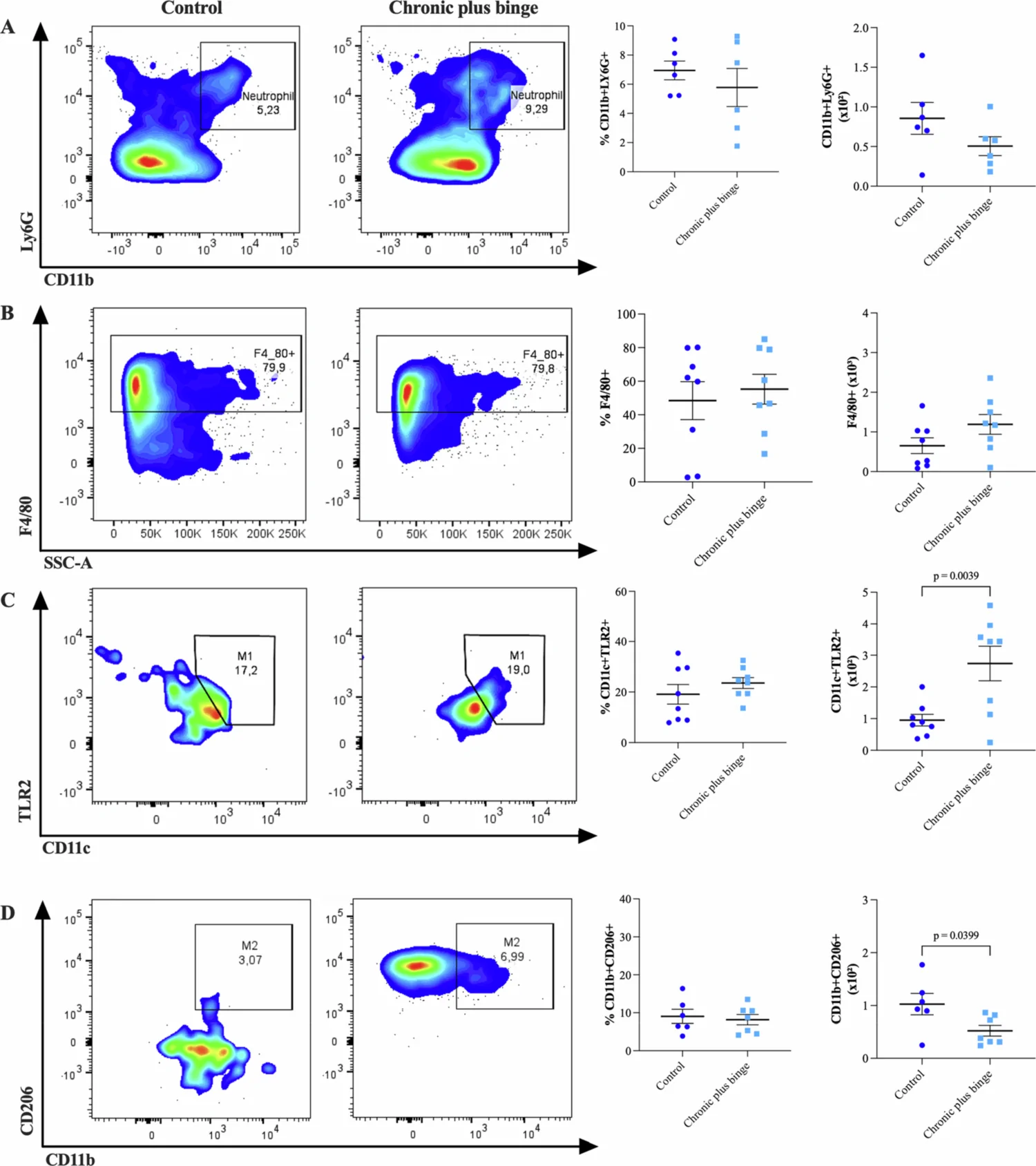
Chronic plus binge ethanol exposure shifts macrophage polarization in abdominal PVAT toward a pro-inflammatory profile. (A–D) Flow cytometric analysis of innate immune populations in PVAT: neutrophils (CD11b^+^Ly6G^+^; *n* = 6/group) (A), total macrophages (F4/80^+^; *n* = 8/group) (B), M1 macrophages (CD11c^+^TLR2^+^; *n* = 8/group) (C), and M2 macrophages (CD11b^+^CD206^+^; *n* = 6-7/group) (D). Left, representative dot plots. Right, quantification of frequency and absolute cell numbers. Each *n* represents pooled PVAT from three mice. Data were obtained from two independent experimental cohorts. Data are presented as mean ± SEM. Statistical analyzes were performed using unpaired Student’s *t*-test (*p* < 0.05; exact *p* values are indicated). Abbreviations: PVAT, perivascular adipose tissue.

In abdominal PVAT, no significant changes were observed in the frequency or absolute number of neutrophils and macrophages between groups (Figure 3A-B). However, ethanol exposure significantly increased the absolute number of M1 macrophages while reduced M2 macrophages, without affecting their relative frequencies (Figure 3C-D). These findings indicate a shift toward a pro-inflammatory macrophage profile in PVAT.

**Figure 4.**
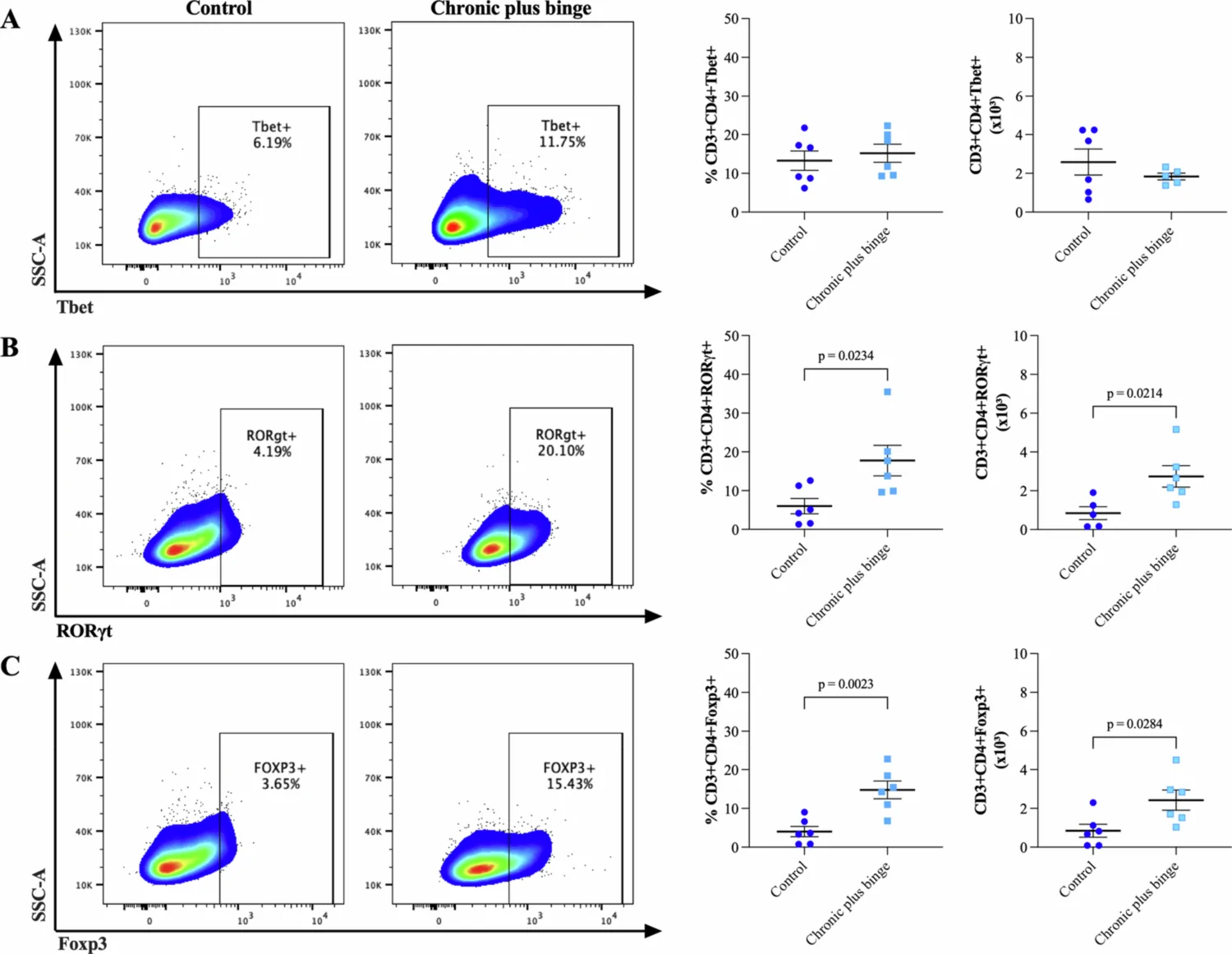
Chronic plus binge ethanol exposure increases Th17 and Treg populations in the cecal lymph node. (A–C) Flow cytometric analysis of Th1 (CD3^+^CD4^+^Tbet^+^; *n* = 5-6/group) (A), Th17 (CD3^+^CD4^+^RORɣt^+^; *n* = 5-6/group) (B), and Treg cells (CD3^+^CD4^+^FoxP3^+^; *n* = 6/group) (C). Left, representative dot plots. Right, quantification of frequency and absolute cell numbers. Data were obtained from one independent experimental cohort. Data are presented as mean ± SEM. Statistical analyzes were performed using unpaired Student’s *t*-test (*p* < 0.05; exact *p* values are indicated). Abbreviations: Th, T helper; Treg, regulatory T.

To assess adaptive immune cells within gut-associated lymphoid tissue (GALT), the largest lymphoid organ in the body,^56^ cecal lymph nodes were analyzed. Ethanol exposure did not affect Th1 cell populations (Figure 4A) but significantly increased both the frequency and absolute number of Th17 and regulatory T (Treg) cells (Figure 4B-C), suggesting concurrent activation of pro-inflammatory and compensatory regulatory immunological pathways within the intestinal immune compartment.

Inflammatory mediators were further evaluated in the AA and PVAT. Chronic plus binge ethanol exposure was associated with a significant reduction in pro-inflammatory cytokine levels in the AA, including TNF-*α*, IL-1β, IL-6, IL-17A, and IL-22 (Table 1). In contrast, IL-1β, IL-17A, and IL-22 levels were significantly increased in PVAT from ethanol-treated mice, while TNF-*α* and IL-6 remained unchanged (Table 1).

Taken together, these findings reveal a compartment-specific immune response characterized by suppression of inflammatory response in the vascular wall alongside a localized Th17 pro-inflammatory environment in PVAT.

### 3.8. Th17/IL-17RA and TLR4 signaling are involved in PVAT dysfunction

Given the expansion of Th17 cells (Figure 4B), alongside increased IL-17A and IL-22 levels in PVAT (Table 1), we next investigated whether Th17-associated signaling contributes to ethanol-induced vascular and PVAT dysfunction. To address this question, Rag1^-/-^ mice, which lack mature B and T cells, and IL-17RA^-/-^ mice were used.

**Figure 5.**
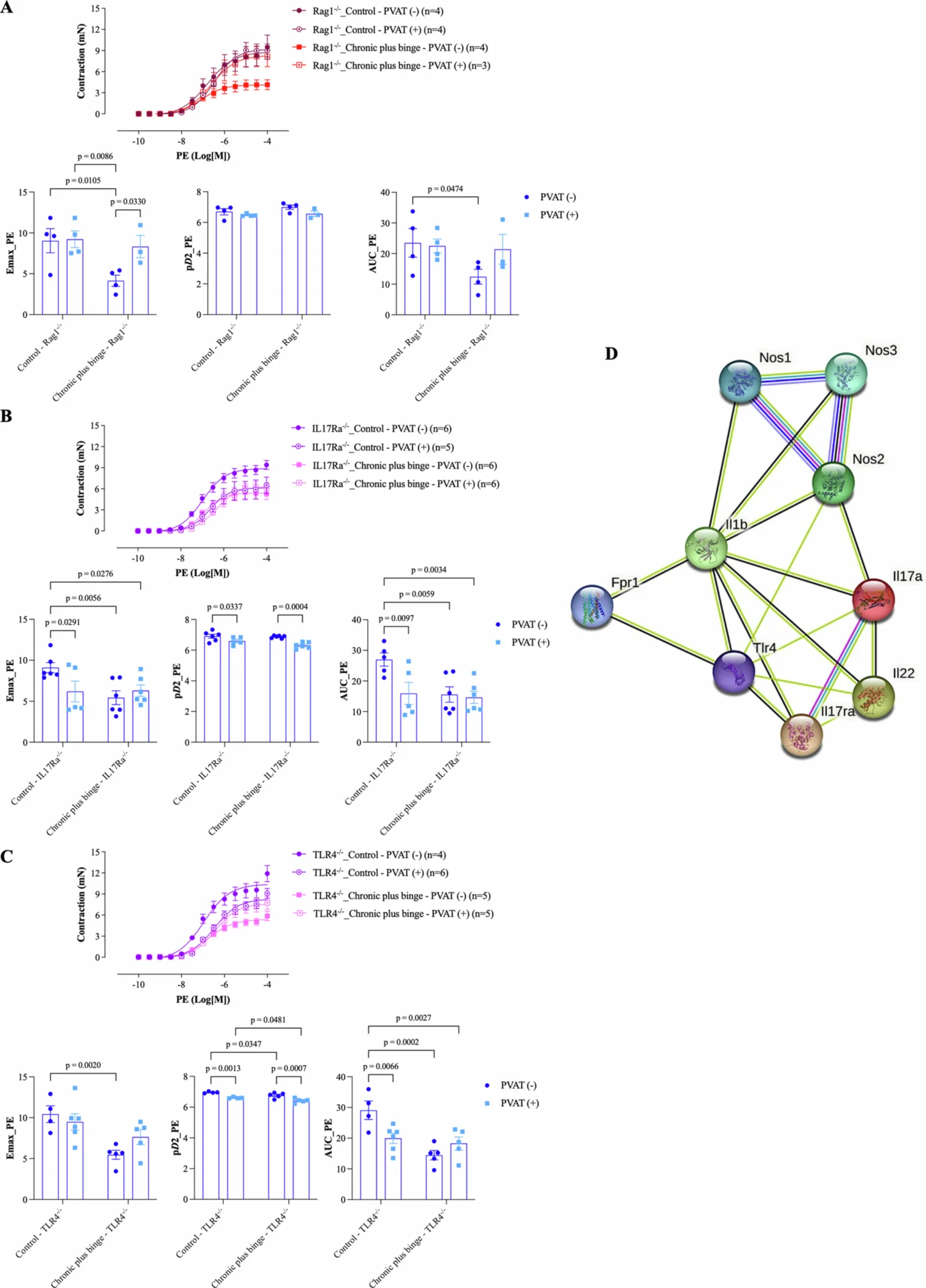
Th17/IL-17RA and TLR4 signaling contribute to PVAT dysfunction induced by chronic plus binge ethanol exposure. (A–C) Concentration–response curves to PE in AA segments with or without PVAT [PVAT (+) or PVAT (–), respectively] from Rag1^-/-^ (*n* = 3-4/group) (A), IL-17RA^-/-^ (*n* = 5-6/group) (B), and TLR4^-/-^ mice (*n* = 4-6/group) (C). Upper, representative curves. Lower, summary data showing E_max_, p*D*_2_, and AUC, respectively. (D) Protein-protein interaction (PPI) network derived from STRING database. Edges indicate both functional and physical protein associations. Data for vascular reactivity assays were obtained from multiple experimental sessions (typically 2 animals per session) to achieve the final sample size under consistent experimental conditions. Sample sizes vary due to technical limitations and the limited availability of transgenic mice. Data are presented as mean ± SEM. Statistical analyzes were performed using two-way ANOVA followed by uncorrected Fisher’s LSD test for multiple comparisons (*p* < 0.05; exact *p* values are indicated). Abbreviations: AA, abdominal aorta; AUC, area under the curve; E_max_, maximum response; IL-17RA^-/-^, IL-17 receptor subunit A knockout (IL-17RA^-/-^); p*D*_2_, -log EC_50_; PE, phenylephrine; PVAT, perivascular adipose tissue; Rag1^-/-^, recombination-activating gene-1 knockout; TLR4^-/-^, Toll-like receptor 4 knockout.

Ethanol-induced vascular hyporesponsiveness in AA-PVAT (–) segments was observed in both Rag1^-/-^ and IL-17RA^-/-^ mice, indicating that this vascular phenotype occurs independently of Th17/IL-17RA signaling. In contrast, ethanol-induced PVAT dysfunction was absent, as no significant differences in contractile responses were observed between AA-PVAT (+) segments from control and ethanol-treated Rag1^-/-^ and IL-17RA^-/-^ mice (Figure 5A-B).

In IL-17RA^-/-^ mice, abdominal PVAT exhibited an anti-contractile phenotype under basal conditions (Figure 5B), whereas this effect was not observed in Rag1^-/-^ mice (Figure 5A). Notably, IL-17RA^-/-^ mice failed to develop the ethanol-induced pro-contractile PVAT phenotype (Figure 5B).

Considering the role of innate immune detection in identifying gut microbiota-derived signals,^11^ we also assessed the contribution of TLR4 to a dysfunctional PVAT in response to ethanol. AA-PVAT (–) segments from ethanol-treated TLR4^-/-^ mice also exhibited hyporesponsiveness, whereas contractile responses in AA-PVAT (+) segments were similarly to those of controls (Figure 5C). Moreover, consistent with data from IL-17RA^-/-^ mice, PVAT from TLR4^-/-^ control mice displayed a basal anti-contractile phenotype (Figure 5B-C).

Collectively, these findings demonstrate that ethanol-induced vascular hyporesponsiveness is independent of immune response signaling, while PVAT dysfunction requires both adaptive (Th17/IL-17RA) and innate (TLR4) immune pathways. These results identify PVAT as a critical immune-metabolic interface through which gut microbiota-driven immune signals promote vascular dysfunction.

### 3.9. Integration of signaling pathways

To explore potential interactions between immune and microbiota-derived signaling pathways, an exploratory PPI network analysis was performed. The resulting network showed significant enrichment (PPI enrichment *p* value = 1.26 × 10^−10^) and revealed predicted interactions among FPR1, IL-17RA, TLR4, and inflammatory mediators, such as IL-1β, IL-17A, IL-22, as well as constitutive and inducible nitric oxide synthase (NOS) enzymes (Figure 5D). Notably, IL-1β emerged as a central node linking these pathways. Importantly, this PPI analysis was used to support the biological plausibility and pathway-level integration of the observed phenotypes, rather than to infer direct molecular interactions or causality.

### 3.10. Redox imbalance and mitochondrial dysfunction are associated with vascular and PVAT dysfunction

**Figure 6.**
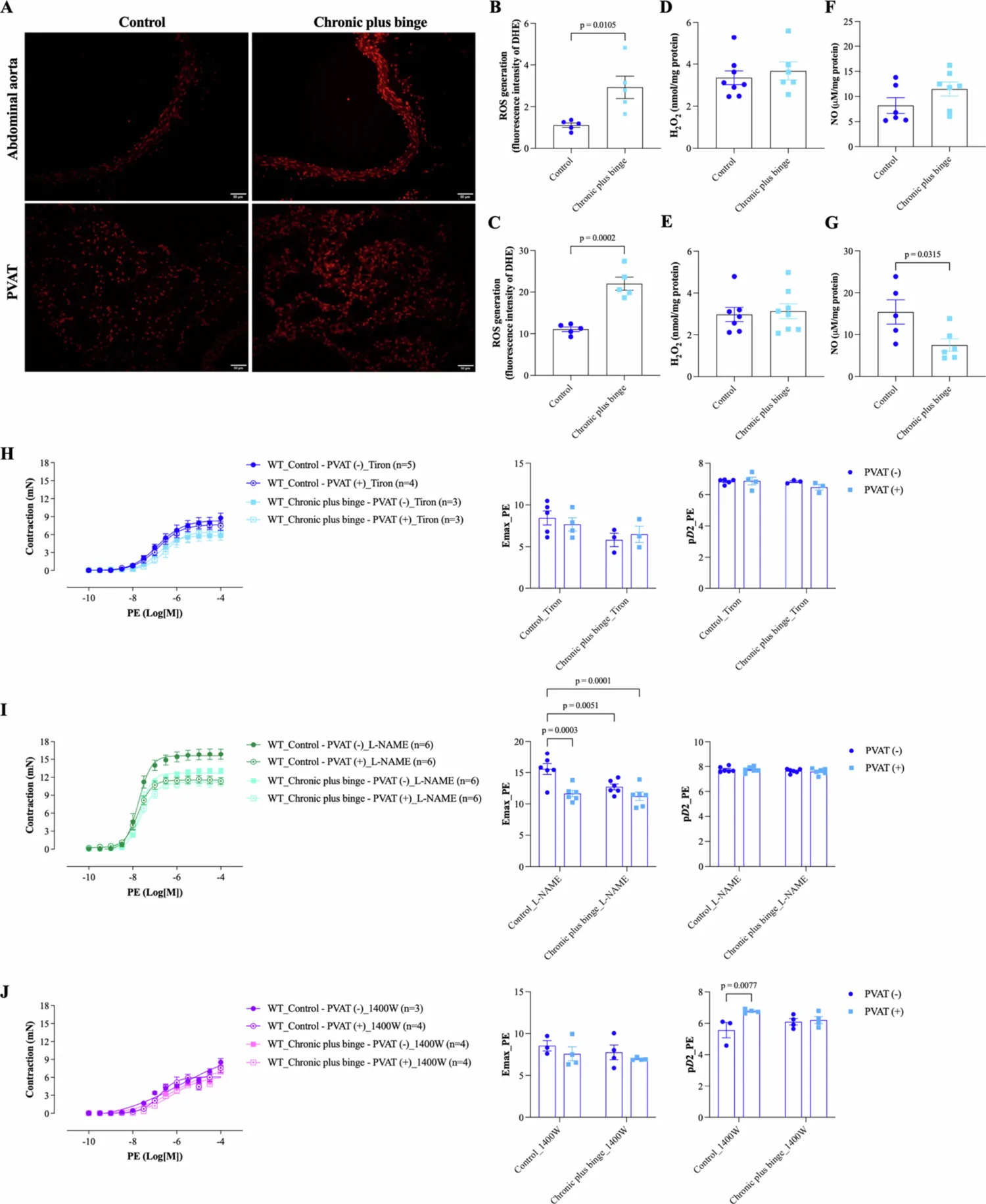
Redox imbalance contributes to AA and PVAT dysfunction induced by chronic plus binge ethanol exposure in male WT mice. (A) Representative fluorescence images of ROS in the AA (top) and PVAT (bottom) using the DHE probe (1 μmol/L; scale bar, 50 μm) in the control and ethanol groups (one representative animal per group). (B-C) Semi-quantitative analysis of the fluorescence intensity of DHE in the AA (B) and PVAT (C), reflecting oxidative stress (*n* = 5/group). (D-E) H_2_O_2_ concentrations in the AA (D) and PVAT. (E) determined by fluorometric assay in the control and ethanol groups (*n* = 6-8/group). (F-G) NO concentrations in the AA (F) and PVAT (G) from control and ethanol-treated mice determined by a Sievers NO Analyzer (*n* = 5-7/group). Data were obtained from one (A–C) or two (D–G) independent experimental cohorts. (H–J) Concentration–response curves to PE in AA segments with or without PVAT [PVAT (+) or PVAT (–), respectively] from WT control and ethanol-treated mice in the presence of O_2_^•−^ scavenger Tiron (100 µmol/L; *n* = 3-5/group) (H), non-selective NOS inhibitor L-NAME (100 µmol/L; *n* = 6/group) (I), or the specific iNOS inhibitor 1400 W (1 µmol/L; *n* = 3-4/group) (J). Left, representative curves. Right, summary data showing E_max_ and p*D*_2_, respectively. Vascular reactivity data were obtained from multiple experimental sessions (typically two animals per session) to achieve the final sample size under consistent experimental conditions. Sample sizes vary due to technical limitations inherent to each assay. Data were obtained from one (A–C) or two (D–G) independent experimental cohorts. Data are presented as mean ± SEM. Statistical analyzes were performed using unpaired Student’s *t*-test (B–E, G), Mann-Whitney test (F), or two-way ANOVA followed by uncorrected Fisher’s LSD test for multiple comparisons (H–J) (*p* < 0.05; exact *p* values are indicated). Abbreviations: AA, abdominal aorta; DHE, dihydroethidium; E_max_, maximum response; H_2_O_2_, hydrogen peroxide; iNOS, inducible nitric oxide synthase enzyme; L-NAME, Nω-Nitro-L-arginine methyl ester; NO, nitric oxide; NOS, nitric oxide synthase enzyme; O_2_^•−^, superoxide anion; p*D*_2_, -log EC_50_; PE, phenylephrine; PVAT, perivascular adipose tissue; ROS, reactive oxygen species.

Having identified gut microbiota- and immune-dependent signaling pathways, we next investigated downstream mechanisms underlying vascular and PVAT dysfunction. ROS generation was significantly increased in both AA and PVAT (Figure 6A–C), whereas H_2_O_2_ levels were unchanged (Figure 6D-E). While NO levels were unchanged in the AA (Figure 6F), a significant reduction was observed in abdominal PVAT from ethanol-treated mice (Figure 6G).

To assess the functional relevance of oxidative stress, *ex vivo* pharmacological interventions were performed. Scavenging of O_2_^•−^ with Tiron restored contractile responses in AA-PVAT (–) segments and normalized PVAT-dependent modulation of vascular tone (Figure 6H), demonstrating a central role for ROS in both vascular and PVAT dysfunction. Inhibition of NOS with L-NAME increased PE-induced contraction in AA-PVAT (–) and AA-PVAT (+) segments from control and ethanol-treated mice (Supplementary Figure S11A-B), consistent with the well-established vasodilatory role of NO. However, ethanol-induced vascular hyporesponsiveness in AA-PVAT (–) persisted following NOS inhibition (Figure 6I). Notably, non-selective NOS inhibition unmasked an anti-contractile PVAT phenotype in both groups (Supplementary Figure S11A-B), suggesting that NO contributes to basal PVAT tone regulation.

To further dissect the contribution of NOS isoforms, the selective iNOS inhibitor 1400 W was employed. Inhibition of iNOS attenuated vascular hyporesponsiveness and PVAT-dependent effects (Figure 6J), supporting that iNOS-derived reactive species contribute to ethanol-induced vascular and PVAT dysfunction. Notably, the pro-contractile PVAT phenotype observed in ethanol-treated mice was abolished following *ex vivo* treatment with Tiron, L-NAME, or 1400 W (Figure 6H–J), reinforcing the involvement of redox-dependent mechanisms.

In this context, a NO-mediated vasoconstrictor effect has been associated with increased peroxynitrite (ONOO^-^) generation through the reaction between NO and O_2_^•−^.^57^ Although ONOO^-^ was not directly measured, its formation was suggested by the coexistence of increased ROS (Figure 6A, C) and reduced NO bioavailability in PVAT from ethanol-treated mice (Figure 6G). Consistently, O_2_^•−^ scavenging prevented ethanol-induced PVAT-dependent alterations, whereas no effects were observed under basal conditions (Figure 6H).

**Figure 7.**
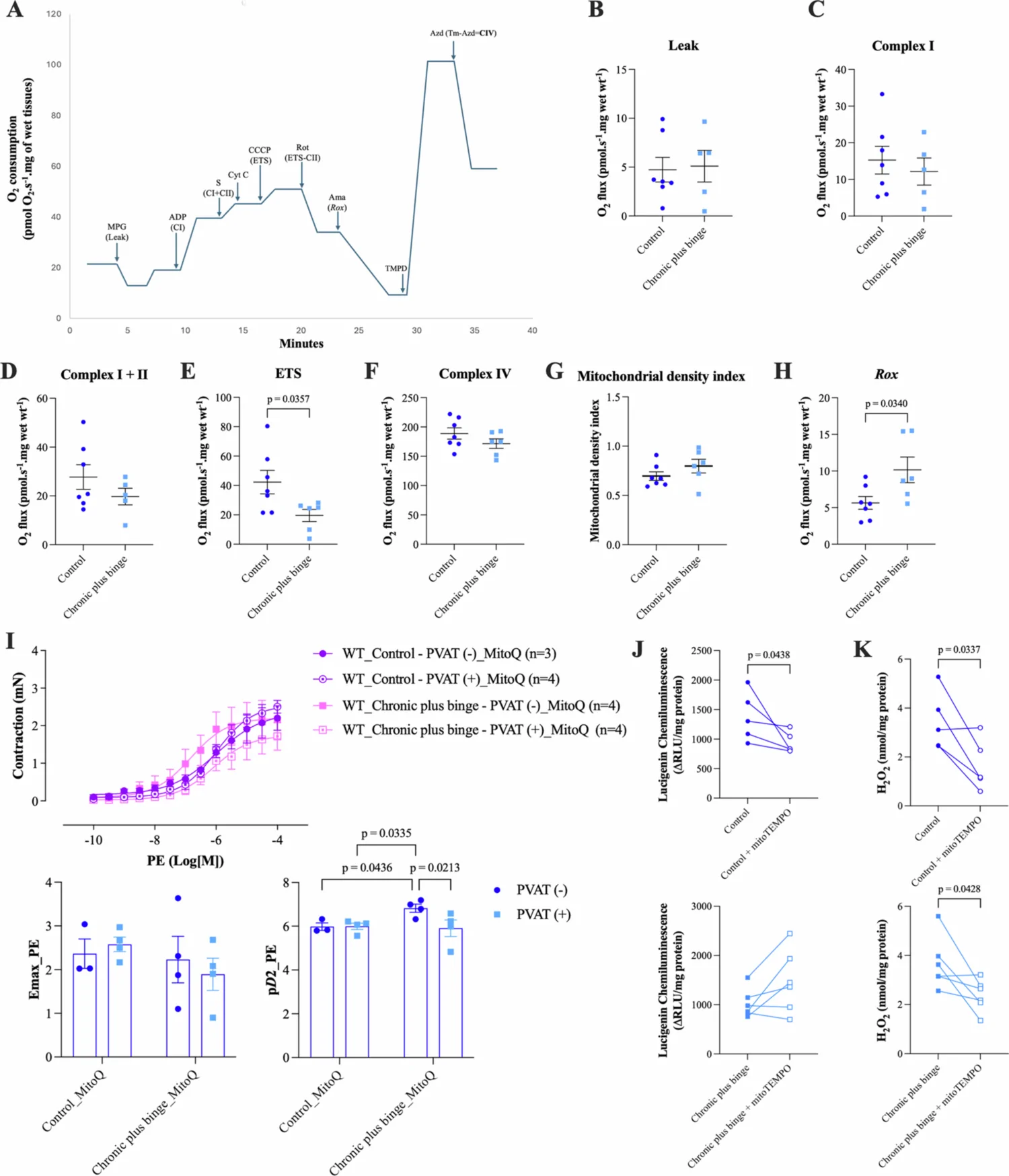
Mitochondrial-derived H_2_O_2_ contributes to vascular hyporesponsiveness induced by chronic plus binge ethanol exposure in male WT mice. (A) Representative trace of oxygen consumption rate (OCR) during sequential addition of mitochondrial substrates and inhibitors. (B–H) Mitochondrial respiration parameters in the AA from WT mice assessed by high-resolution respirometry (*n* = 5-7/group). The following parameters were determined: leak respiration (B), CI activity (C), combined CI+CII activity (D), maximum capacity of the ETS (E), CIV activity (F), mitochondria density index (G), and extra-mitochondrial oxygen consumption (*Rox* index) (H). Data were obtained from three independent experimental cohorts. (I) Concentration–response curves to PE in AA segments with or without PVAT [PVAT (+) or PVAT (–), respectively] in the presence of the mitochondria-targeted antioxidant MitoQ (100 nmol/L; *n* = 3-4/group) in control and ethanol-treated mice. Upper, representative curves. Lower, summary data showing E_max_ and p*D*_2_, respectively. Vascular reactivity data were obtained from multiple experimental sessions (typically two animals per session) to achieve the final sample size under consistent experimental conditions. (J–K) O_2_^•-^ (J) and H_2_O_2_ (K) levels in the AA from control (top) or ethanol-treated (bottom) mice in the presence of vehicle or the mitochondria-targeted antioxidant mitoTEMPO (100 nmol/L), determined by lucigenin chemiluminescence or Amplex Red assays, respectively (*n* = 5-6/group). Data were obtained from two independent experimental cohorts. Sample sizes vary due to technical limitations. Data are presented as mean ± SEM. Statistical analyzes were performed using unpaired (B–H) or paired (J–K) Student’s *t*-test, or two-way ANOVA followed by uncorrected Fisher’s LSD test for multiple comparisons (I) (*p* < 0.05; exact *p* values are indicated). Abbreviations: AA, abdominal aorta; ADP, adenosine diphosphate; CI, complex I activity; CII, complex II activity; CIV, complex IV activity; Cyt C, cytochrome C; E_max_, maximum response; ETS, electron transfer system; H_2_O_2_, hydrogen peroxide; Leak, index of proton leak; MitoQ, mitoquinone; MPG, malate, pyruvate, and glutamate; OCR, oxygen consumption rate; p*D*_2_, -log EC_50_; PE, phenylephrine; PVAT, perivascular adipose tissue; Rot, rotenone; *Rox*, residual oxygen consumption; S, succinate; TMPD, *N*-N-N′-N′-Tetramethyl-p-phenylenediamine dihydrochloride.

Given the established link between ethanol exposure and mitochondrial dysfunction,^8^ we next evaluated mitochondrial function in the AA (Figure 7A–H). Ethanol exposure impaired mitochondrial respiration, as evidenced by reduced maximal electron transport system capacity (Figure 7E). In addition, ethanol-treated mice exhibited a significant increase in extra-mitochondrial oxygen utilization (Figure 7H), consistent with disrupted cellular redox homeostasis. Notably, *ex vivo* treatment with the mitochondria-targeted antioxidant MitoQ restored the contractile response in AA-PVAT (–) segments and attenuated PVAT-dependent differences between groups (Figure 7I). Consistently, comparisons restricted to AA-PVAT (–) segments reinforced that attenuation of mitochondrial ROS (mt-ROS) mitigates ethanol-induced vascular tone impairment (Supplementary Figure 12A). In AA-PVAT (+) segments, although MitoQ attenuated vasocontractility differences between control and ethanol-treated mice, no differences were observed in the contractile responses within ethanol group when comparing basal to MitoQ-conditioned states (Supplementary Figure 12B). These findings suggest that mt-ROS contribute to ethanol-induced hypocontractility, but are unlikely to underlie the pro-contractile PVAT phenotype observed in ethanol-treated mice.

To further investigate the specific mt-ROS involved in ethanol-induced vascular effects, O_2_^•-^ and H_2_O_2_ levels were measured in the AA following incubation with the mitochondria-targeted antioxidant mitoTEMPO. In control mice, mitoTEMPO decreased both O_2_^•-^ and H_2_O_2_ levels (Figure 7J–K), suggesting a role for mt-ROS in maintaining physiological redox balance. In contrast, in ethanol-treated mice, mitoTEMPO significantly reduced H_2_O_2_ levels, without affecting O_2_^•-^ levels (Figure 7J–K). Taken together, these results suggest that mitochondria-derived H_2_O_2_, along with ROS from additional sources, contributes to ethanol-induced vascular dysfunction.

**Figure 8.**
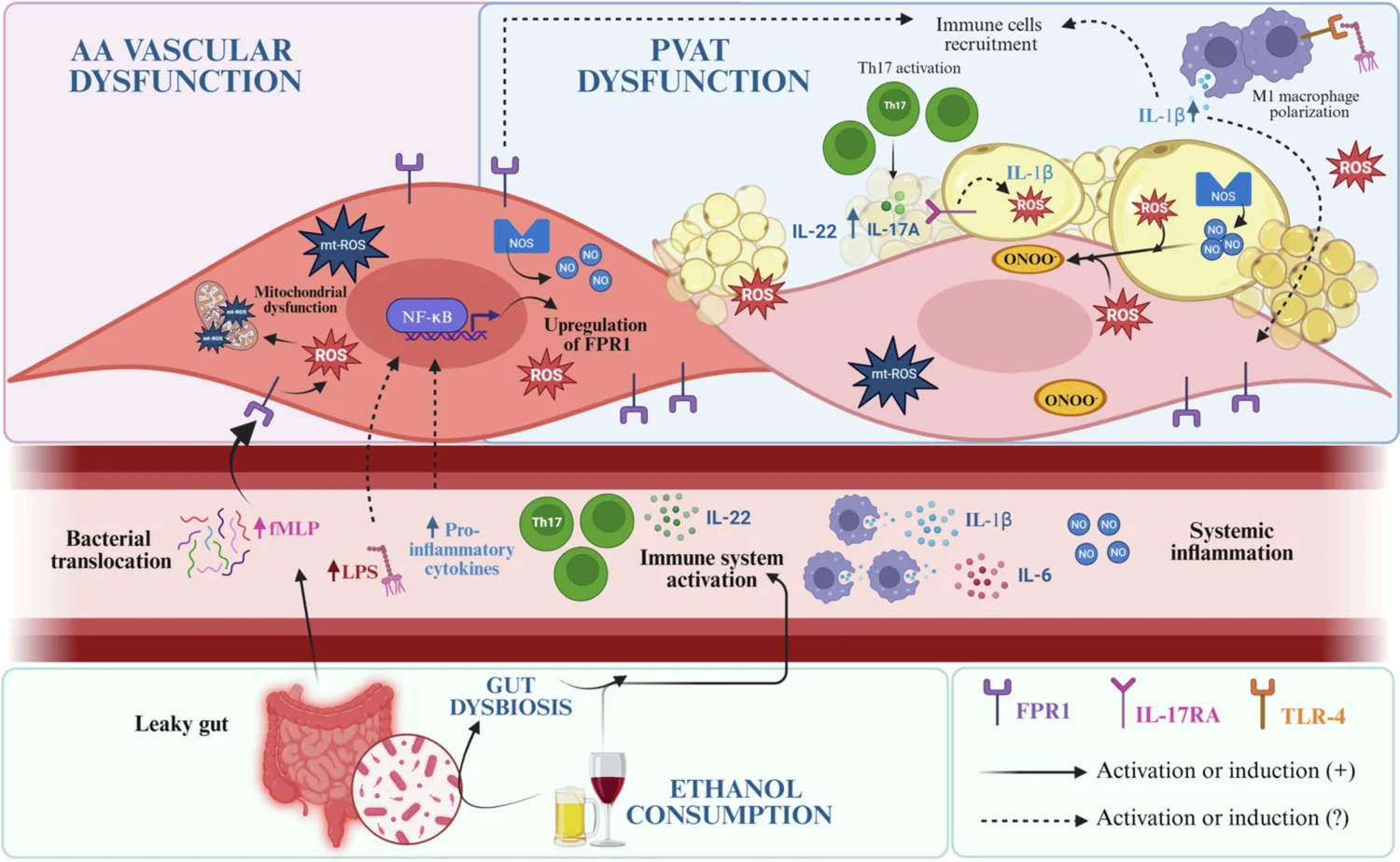
Proposed mechanisms linking gut dysbiosis to ethanol-induced AA and PVAT dysfunction. Chronic plus binge ethanol exposure induces gut dysbiosis and disrupts intestinal barrier integrity, increasing systemic levels of microbiota-derived products (i.e., LPS and fMLP). This is accompanied by systemic immune activation, reflected by increased levels of NO and pro-inflammatory cytokines (i.e., IL-1β, IL-6, and IL-22), potentially mediated by gut dysbiosis and/or direct ethanol-induced modulation of the immune system. Upregulation of FPR1, mediated by cytokines and/or LPS, enhances responsiveness to fMLP, promoting oxidative stress, mitochondrial dysfunction, and mt-ROS generation. FPR1 activation may also facilitate immune cell recruitment and iNOS-derived NO production, further contributing to AA hypocontractility. In parallel, PVAT dysfunction is associated with M1 polarization and Th17 expansion, resulting in a pro-inflammatory profile with increased levels of IL-1β, IL-17A, and IL-22. Activation of IL-17RA and TLR4 signaling further amplifies nitro-oxidative stress and may favor ONOO^-^ formation, driving a pro-contractile PVAT phenotype in ethanol-treated mice. These findings support a gut–immune–PVAT axis linking microbiota-derived signals to ethanol-induced vascular dysfunction. Created in BioRender. Abbreviations: AA, abdominal aorta; fMLP, N-formyl–methionyl–leucyl–phenylalanine; FPR1, formyl peptide receptor 1; IL, interleukin; iNOS, inducible nitric oxide synthase; LPS, lipopolysaccharide; mt-ROS, mitochondrial reactive oxygen species; NO, nitric oxide; ONOO^-^, peroxynitrite; PVAT, perivascular adipose tissue; ROS, reactive oxygen species; TLR4, Toll-like receptor 4; Th, T helper.

The proposed mechanisms underlying AA and PVAT dysfunction induced by chronic plus binge ethanol exposure are summarized in Figure 8.

## 4. Discussion

Our findings extend the current understanding of ethanol-induced pathology by establishing a novel gut–immune–PVAT axis. While emerging evidence has identified gut dysbiosis as a key mediator of ethanol-associated injury,^5^ ^,^ ^8^ ^,^ ^32^ ^,^ ^35^ ^,^ ^58-60^ our prior study showed that ethanol alters the gut microbiome in a pattern- and sex-dependent manner.^6^ Herein, we demonstrate that gut microbiota disruption induced by chronic plus binge ethanol exposure is accompanied by bacterial translocation, elevation in circulating levels of microbiota-derived products (i.e., LPS and fMLP), and activation of both innate and adaptive immunological responses. Consistently, our data demonstrate that this paradigm synergistically exacerbates systemic inflammation, as evidenced by increased levels of the pro-inflammatory markers (IL-1β, IL-6, IL-22 and NO), exceeding the effects observed with either chronic or binge exposure alone in male WT mice.

Distinct transgenic mouse models (FPR1^fl/fl^/Myh11-Cre, Rag1^-/-^, IL-17RA^-/-^, and TLR4^-/-^) were utilized to evaluate complementary arms of this microbiota–immune–vascular signaling cascade. From a mechanistic perspective, our findings support that PVAT acts as a dynamic sensor of microbiota-derived signals under pathological conditions. In this context, PVAT integrates microbial, immunological, and metabolic cues into functional vascular responses. This framework suggests that targeting PVAT-specific pathways –or upstream microbiota-derived signals– may represent a more effective strategy than approaches focused exclusively on the vascular wall. Importantly, while our data provide mechanistic insights involving FPR1, TLR4, and Th17/IL-17RA signaling, a definitive dissection of these interconnected pathways warrants further investigation.

Clinical studies have suggested a link between ethanol consumption and abdominal aortic aneurysm (AAA), a severe vascular disease characterized by progressive aortic dilatation.^61^ Although no alterations in vascular diameter were found in our model, our findings demonstrated that ethanol induced significant vascular remodeling, as evidenced by reduced collagen content and altered stress-strain curve. These changes suggest increased vascular distensibility and reduced structural stiffness, which are known precursors to aneurysm formation and rupture risk. Increased ROS production is known to promote VSMC proliferation and remodeling, thereby contributing to cardiovascular risk.^20^ PVAT plays a central role in regulating redox balance and inflammation through its interaction with endothelial cells, VSMC, and immune cells,^21^ and PVAT dysfunction has been implicated in abdominal aortic diseases.^62^ ^,^ ^63^

Consistent with previous reports of multi-organ injury induced by chronic plus binge ethanol exposure,^7^ ^,^ ^8^ ^,^ ^24^ ^,^ ^32^ ^,^ ^34-37^ ^,^ ^64^ our data demonstrated end-organ damage, including spleen and kidney atrophy, along with a compartment-specific vascular phenotype. This phenotype is characterized by reduced contractile responses in the AA and shift of abdominal PVAT toward a pro-inflammatory and dysfunctional state. This includes M1 macrophages polarization, elevated levels of IL-1β, IL-17A, and IL-22, and nitro-oxidative stress, evidenced by increased ROS generation along with decreased NO bioavailability. Notably, PVAT dysfunction was associated with increased bacterial DNA content, supporting the occurrence of bacterial translocation, and with an increased Bacillota to Bacteroidota ratio, a feature previously linked to alcoholic liver disease.^65^ Our data using GF models demonstrated that the gut microbiota is a fundamental determinant of ethanol-induced alterations in intestinal barrier integrity, renal injury, and abdominal PVAT dysfunction.

Contrasting with previous reports that described enhanced vascular reactivity following ethanol exposure,^16^ ^,^ ^17^ ^,^ ^66^ we observed vascular hyporesponsiveness. In our model, no changes in relaxation responses were observed. However, impaired endothelium-dependent relaxation has been reported in mice subjected to chronic alcohol consumption for 20 d, in addition to two binges of alcohol every tenth day.^7^ Discrepancies across studies likely reflect differences in ethanol exposure patterns, duration, and vascular beds analyzed.

Oxidative stress is a central mechanism underlying ethanol-induced vascular impairment and hypertension.^67^ In addition, mitochondrial dysfunction has been implicated in ethanol-induced cardiovascular injury.^8^ ^,^ ^17^ Consistent with these observations, ethanol-induced functional and structural alterations in the AA were associated with increased ROS generation and mitochondrial dysfunction, further amplifying redox imbalance. Notably, ethanol-induced alterations in vascular tone and PVAT dysfunction were attenuated by *ex vivo* treatment with both a O_2_^•−^ scavenger and a selective mt-ROS scavenger.

Our findings using mitoTEMPO suggest that mitochondria-derived H_2_O_2_, rather than O_2_^•−^, is linked to ethanol-induced vascular dysfunction. While H_2_O_2_ can act as either a vasorelaxing or vasoconstrictor factor depending on its concentration,^67^ our data support its role in the observed impairment. Furthermore, mitochondria are susceptible to oxidative damage, and oxidized mitochondrial DNA can act as a damage-associated molecular pattern (DAMP), further promoting inflammation.^68^ Taken together, these data indicate that mitochondria-derived H_2_O_2_, along with ROS from additional sources, contributes significantly to the vascular effects of chronic plus binge ethanol exposure.

While nonselective NOS inhibition did not restore vascular hyporesponsiveness, selective iNOS inhibition attenuated both vascular and PVAT dysfunction, suggesting a central role for iNOS-derived NO in ethanol-induced alterations. Although ONOO^-^ formation was not directly measured, the biochemical context supports its involvement. Specifically, the coexistence of increased ROS and reduced NO bioavailability, together with the reversal of dysfunction by ROS scavenging and iNOS inhibition, is consistent with a model in which nitro-oxidative stress drives PVAT functional remodeling.

While microbiota-derived metabolites, such as TMAO, have been implicated in vascular dysfunction,^9^ no changes in circulating TMAO levels were observed in this study. In contrast, ethanol exposure increased systemic levels of fMLP and upregulated vascular FPR1 expression, potentially enhancing responsiveness to this ligand. FPR1 activation triggers intracellular signaling pathways involved in chemotaxis, ROS production, cytokine release, and cytoskeletal remodeling.^14^ ^,^ ^69^ Interestingly, while pharmacological inhibition of FPR1 did not revert AA hyporesponsiveness, this effect was prevented in FPR1^-/-^ mice. Moreover, the pro-contractile PVAT phenotype was mitigated following inhibition with CsH, and the SMC-specific deletion of FPR1 was associated with an anti-contractile effect induced by ethanol. These discrepancies may reflect differences in receptor localization, signaling compartmentalization, or ligand-specific activation. FPRs have been described not only at the plasma membrane but also in intracellular compartments, including nucleus and secretory vesicles.^70^ In addition, biased agonism –dependent on ligand concentration and exposure time– may result in distinct downstream signaling pathways.^70^ ^,^ ^71^ These mechanisms may explain the differential effects observed between pharmacological and genetic approaches.

In the absence of the gut microbiota, FPR1 may respond to endogenous ligands (e.g., cathepsin G and annexin A1) or DAMPs (e.g., mitochondrial-derived peptides), eliciting distinct signaling responses.^69^ PPI network analysis supported interactions between FPR1, TLR4, and IL-1β. LPS-mediated activation of TLR4 and inflammasome pathways represents a key mechanism linking gut-derived signals to systemic inflammation.^72^ Although IL-1β has been implicated in ethanol-induced tissue injury,^73^ its specific role in PVAT dysfunction remains to be determined.

Corroborating our findings, microbiota-derived signals contribute to immune activation in both systemic circulation and peripheral tissues.^72^ Immune cells within PVAT can release effector cytokines that promote oxidative stress and vascular dysfunction.^52^ In particular, M1 macrophage polarization and Th17 cell expansion were observed following ethanol exposure, along with increased Treg cells, possibly reflecting compensatory immune regulation. These findings align with previous reports linking gut dysbiosis to modulation of T cell responses.^59^

The functional relevance of this immune landscape is supported by the loss of PVAT-dependent effects in Rag1^-/-^, IL-17RA^-/-^, and TLR4^-/-^ models, indicating that both adaptive and innate pathways contribute to the full manifestation of PVAT dysfunction. However, whether these phenotypes are independent or mechanistically linked remains to be elucidated. Notably, vascular hyporesponsiveness in PVAT-denuded vessels was unaffected in these models, suggesting that immune signaling primarily targets PVAT rather than the vascular wall.

Under basal conditions, abdominal PVAT did not exhibit a significant anti-contractile effect in WT mice, consistent with regional heterogeneity of PVAT function.^53^ In contrast, GF mice displayed a clear anti-contractile phenotype associated with higher basal FPR1 expression within PVAT, suggesting that microbiota-derived signals modulate abdominal PVAT function. While alterations in FPR1 signaling may contribute to these differences, this pathway appears to be less dominant in the presence of a normal microbiota. Additionally, IL-17RA and TLR4 signaling were found to modulate abdominal PVAT function under physiological conditions, further supporting the role of PVAT as na interface between the gut microbiota-immune axis and vascular homeostasis.

Although reduced NO bioavailability has been associated with the lack of a basal anti-contractile phenotype in the abdominal PVAT,^53^ our findings suggest a more complex, context-dependent role for NO signaling. Notably, pharmacological inhibition of NOS with L-NAME, but not with 1400 W, unmasked an anti-contractile effect of the abdominal PVAT. This suggests that constitutive NOS isoforms **–**rather than inducible ones**–** are involved in a complex regulatory crosstalk where downstream signaling pathways or compensatory mechanisms, rather than NO availability *per se*, may be the critical determinants of abdominal aortic vascular tone. The mechanisms underlying this paradoxical effect, possibly involving an interplay between NO and other PVAT-derived relaxing factors (PVRFs), warrant further investigation.

Importantly, the ethanol exposure paradigm used in the present study reflects clinically relevant patterns of high-risk alcohol consumption in humans (>7 drinks/week for women and >14 drinks/week for men) and supports the presence of an early, preclinical vascular injury state. The absence of changes in blood pressure is consistent with this interpretation. These early alterations in PVAT and vascular function may predispose to CVD progression and represent potential therapeutic targets. Future studies extending ethanol exposure duration or combining this model with additional stimuli (e.g., angiotensin II) will be critical to determine whether these changes progress to overt pathology like hypertension or aneurysm.

## 5. Limitations and future directions

While this study elucidates a novel gut–immune–PVAT axis as a modulator of cardiovascular dysfunction induced by excessive ethanol consumption, we recognize some limitations. Endothelium-independent vascular responses were not assessed; however, a growing body of evidence suggests that endothelium-derived factors mediate ethanol-induced PVAT dysfunction.^16^ Some experiments were performed with limited sample sizes; however, the convergence of findings across pharmacological, genetic, and biochemical approaches strengthens the robustness of our conclusions. Although GF models provide strong evidence for microbiota involvement, they also present developmental and immunological differences. Future studies using fecal microbiota transplantation (FMT), antibiotic depletion, and cell-specific genetic deletions (e.g., LysM-Cre) will be valuable to establish causality and define cell-type-specific mechanisms. Additionally, direct visualization of bacterial translocation into PVAT would strengthen these findings. Sex-specific effects also warrant further investigation.

## 6. Conclusion

Chronic plus binge ethanol exposure is associated with abdominal aortic hyporesponsiveness and PVAT dysfunction within a framework of microbiota-dependent metabolic and immune signaling. Our data support the involvement of fMLP/FPR1, Th17/IL-17A/IL-17RA, and TLR4 pathways and highlight the contribution of gut microbiota-derived and immune-mediated mechanisms to ethanol-induced vascular alterations. In addition, gut microbiota, FPR1, IL-17RA, and TLR4 signaling appear to modulate abdominal PVAT function under physiological conditions. Collectively, these findings support a mechanistic model in which chronic plus binge ethanol exposure engages a gut microbiota–immune–redox axis that converges on PVAT to drive vascular dysfunction. By positioning PVAT as a central integrator of microbiota-derived signals, this study advances the understanding of ethanol-induced cardiovascular injury and identifies potential therapeutic targets within the gut–vascular interface.

## Supplementary Material

R2_Supplementary Material and Methods.pdf R2_Supplementary Material and Methods.pdf

R2_Supplementary Figures.docx R2_Supplementary Figures.docx

R2_Supplementary Tables.docx

## Data Availability

The original contributions presented in the study are included in the article and supplemental material. Further inquiries can be directed to the corresponding author.
