# Supplementary material for: Gut–immune–PVAT axis is involved in ethanol-induced abdominal aortic dysfunction via IL-17RA, TLR4, and FPR1 signaling
Source: Gut Microbes. 2026 Sep 21;18(1):2734648. doi: 10.1080/19490976.2026.2734648 (PMC13596876; doi:10.1080/19490976.2026.2734648)
Supplement: Supplementary file 1 [file KGMI_A_2734648_SM7569.pdf]

Supplementary Material and Methods

Gut–immune–PVAT axis is involved in ethanol-induced abdominal aortic dysfunction via IL-17RA, TLR4, and FPR1 signaling

Carla Brigagão Pacheco da Silva[1,2*]; Vanessa Fernandes Rodrigues[3]; Daniela Carlos[3]; Maria Cecília Jordani[4]; Leandra Naira Zambelli Ramalho[5]; José Teles de Oliveira-Neto[1]; Jefferson Elias-Oliveira[3]; Marília Cristina Oliveira Souza[6]; Rafael Menezes da Costa[1,7]; Mirele Resende Machado[1]; Milene Tavares Fontes[2]; Paul Townsend Jr[2]; Rinaldo Rodrigues dos Passos[2]; Ricardo Bernardino de Paula[2]; Tiago Januário Costa[8]; Cintia Vieira dos Santos[1,2]; Gabriel Henrique Savietto[6]; Daniel Rodrigues[1]; Christiane Becari[4,9]; Cameron G McCarthy[2]; R Clinton Webb[2]; Camilla Ferreira Wenceslau[2]; Rita C Tostes[1].

[1] University of São Paulo, Ribeirao Preto Medical School, Department of Pharmacology, Ribeirão Preto, SP, Brazil.

[2] University of South Carolina School of Medicine, Cardiovascular Translational Research Center, Department of Cell Biology and Anatomy, Columbia, SC, United States.

[3] University of São Paulo, Ribeirão Preto Medical School, Department of Biochemistry and Immunology, Ribeirão Preto, SP, Brazil.

[4] University of São Paulo, Ribeirão Preto Medical School, Department of Surgery and Anatomy, Ribeirão Preto, SP, Brazil.

[5] University of São Paulo, Ribeirão Preto Medical School, Department of Pathology, Ribeirão Preto, SP, Brazil.

[6] University of São Paulo, School of Pharmaceutical Sciences of Ribeirão Preto, Department of Biomolecular Sciences, Ribeirão Preto, SP, Brazil.

[7] Federal University of Jatai, Institute of Health Sciences, Jatai, GO, Brazil.

[8] University of São Paulo, Institute of Biomedical Science, Department of Pharmacology, São Paulo, São Paulo, Brazil.

[9] University of São Paulo, School of Dentistry of Bauru, Department of Biological Sciences, Bauru, São Paulo, Brazil.

*Author for correspondence:
Carla Brigagão Pacheco da Silva

Av. Bandeirantes 3900, Monte Alegre, Ribeirão Preto, 14049900, São Paulo, Brazil. Phone: +551633153181. e-mail: carla.brigagao@alumni.usp.br

Supplementary Material and Methods

1. Material and Methods

1.1. Animals

Male, 8-week-old, C57BL/6J wild type (WT) mice were obtained from either the breeding facility of the Ribeirao Preto Medical School-University of Sao Paulo (FMRP-USP, Ribeirao Preto, SP, Brazil) or The Jackson Laboratory (stock #000664, Bar Harbor, ME, USA). Age-matched female WT mice were obtained from the FMRP-USP facility. Genetically modified strains, including IL-17 receptor subunit A knockout (IL-17RA$^{-/-}$), recombination-activating gene-1 knockout (Rag1$^{-/-}$), and Toll-like receptor 4 knockout (TLR4$^{-/-}$) mice were obtained from the institutional breeding facility at the Ribeirao Preto Medical School. C57BL/6NTac germ-free (GF) mice were generated and maintained under sterile conditions at the University of South Carolina gnotobiotic facility. Smooth muscle cell (SMC)-specific FPR1-deficient (FPR1$^{fl/fl}$/Myh11-CreER^{T2}) mice were generated and maintained at the University of South Carolina School of Medicine. All experimental protocols were approved by the Animal Care and Use Committees of the University of Sao Paulo and the University of South Carolina (CEUA #1007/2021 and IACUC #2596-101690-041122, respectively).

1.2. Genetic loss-of-function models

1.2.1 FPR1$^{fl/fl}$/Myh11-CreER^{T2} mice

Male B6.FVB-Tg(Myh11-icre/ERT2)1Soff/J mice were obtained from The Jackson Laboratories (Bar Harbor, ME, USA). Floxed FPR1 mice (RKIP FPR1$^{fl/fl}$) were generated by BioCytogen (Wakefield, MA, USA) specifically for the laboratory of Dr Camilla Ferreira Wenceslau. Myh11-CreER^{T2} mice were crossed with RKIP FPR1$^{fl/fl}$ mice to generate an inducible, smooth muscle cell (SMC)-specific FPR1 loss-of-function model (FPR1$^{fl/fl}$/Myh11-CreER^{T2}). The Myh11-CreER^{T2} (also referred to as SMMHC-iCreER^{T2}) system allows tamoxifen-inducible Cre recombinase expression selectively in SMCs. A known limitation of this model is that the Cre transgene is located on the Y chromosome, restricting its use to male mice and precluding inclusion of females.[1]

All tamoxifen-related animal procedures were approved by the Animal Care and Use Committee at the University of South Carolina School of Medicine (IACUC #2596-101690-041122). Male, 6-week-old, FPR1$^{fl/fl}$/Myh11-CreER^{T2} mice received tamoxifen-containing chow (Teklad Global 16% Protein Rodent Diet; 250 mg tamoxifen/kg diet) for 7 consecutive days. Based on an average food intake of ~4 g/day, this regimen delivered approximately 40

mg/kg/day of tamoxifen. To ensure efficient and controlled recombination, tamoxifen was administered using a ramped protocol (3 days at half dose followed by 4 days at full dose).

Genotyping of Myh11-CreER^{T2} and FPR1 alleles was performed by PCR using protocols adapted from The Jackson Laboratory (available at: https://www.jax.org/Protocol?stockNumber=019079&protocolID=20071). Primer sequences and expected amplicon sizes are provided in Supplementary Tables S1 and S2, respectively. PCR-based genotyping confirmed recombination efficiency, and prior validation of this model has demonstrated robust deletion of FPR1 in SMCs (Supplementary Fig. S1).

1.2.2 Rag1$^{-/-}$, IL-17RA$^{-/-}$, and TLR4$^{-/-}$ mice

Rag1-, IL-17RA-, and TLR4-deficient mouse strains were bred and maintained under specific pathogen-free (SPF) conditions at the Special Mouse Breeding Center of the Ribeirao Preto Medical School (University of Sao Paulo). The IL-17RA$^{-/-}$ mice (B6.129P2-Il17ra^{tm1Kolls}/J) were originally obtained from TAAM (Typage et Archivage d'Animaux Modèles, CNRS, Orléans, France). The Rag1$^{-/-}$ mice (B6.129S7-Rag1^{tm1Mom}/J) were originally obtained from The Jackson Laboratory (stock #002216). The TLR4$^{-/-}$ mice (Tlr4^{tm1Aki}) were originally generated by Dr. Shizuo Akira (Osaka University, Japan) and were kindly provided via the Federal University of Minas Gerais (UFMG, Belo Horizonte, MG, Brazil).

All strains were maintained on a C57BL/6J genetic background, and strain-matched controls were used for all experimental comparisons. These genetically modified models are well-established and have been extensively validated in previous studies by our group and others to investigate distinct immune pathways, including adaptive immunity (Rag1$^{-/-}$), IL-17 receptor signaling (IL-17RA$^{-/-}$), and innate immune sensing via Toll-like receptor 4 (TLR4$^{-/-}$).[2–6] These models were selected to dissect the complementary immune mechanisms underlying gut microbiota-dependent vascular and inflammatory responses to ethanol exposure.

1.3. Evaluation of vascular function in female mice

Female WT mice were randomly assigned to control or the chronic plus binge ethanol groups, as previously described.[7] Vascular reactivity was assessed in suprarenal segments of the abdominal aorta using concentration-response curves to phenylephrine (PE; 0.1 nmol/L to 100 µmol/L), as detailed in the Materials and Methods section of the main manuscript. These experiments were performed as an initial assessment to determine whether ethanol-induced dysbiosis affects vascular function in females and to inform the design of subsequent mechanistic studies.

1.4. Characterization of ethanol toxicity induced by different alcohol drinking patterns

Male mice were randomly assigned to 4 groups: control, chronic ethanol, binge or chronic plus binge ethanol, as previously described.[7] Intestinal inflammation, intestinal barrier integrity, bacterial translocation and systemic inflammation were evaluated, as detailed in the Materials and Methods section of the main manuscript.

1.5. Statistical Analysis

Data are presented as mean ± standard error of the mean (SEM), with sample sizes (*n*) indicated in the respective figures and tables. Statistical analyses were performed using GraphPad Prism® (version 10.5.0, GraphPad Software Inc., San Diego, CA, USA). Samples were excluded from the final analysis based on pre-established technical criteria, including visible hemolysis, failure to meet quality control thresholds (e.g., RT-qPCR amplification failure), or accidental injury during oral gavage (e.g., FITC-dextran assay). Statistical outliers were identified using the ROUT method (Q = 1%) and excluded where applicable. Data normality was assessed using the Shapiro-Wilk test ($\alpha = 0.05$). For normally distributed data, statistical comparisons were performed using paired or unpaired Student's *t*-tests, one-way ANOVA, or two-way ANOVA, followed by Bonferroni's post hoc test or uncorrected Fisher's least significant difference (LSD) test for multiple comparisons, as appropriate. For non-normally distributed data, the Mann-Whitney test or Kruskal-Wallis test followed by Dunn's post hoc test were employed. Statistical significance was defined as $p < 0.05$. Detailed statistical analysis information is provided in the Supplementary Material.

For vascular reactivity experiments involving two experimental factors (e.g., ethanol treatment and PVAT presence), two-way ANOVA was applied. Fisher's least significant difference (LSD) test was used for predefined pairwise comparisons following detection of significant main or interaction effects, thereby limiting inflation of type I error while preserving statistical sensitivity in the context of biologically constrained sample sizes.

Although paired vascular segments [AA-PVAT (-) and AA- PVAT (+)] were obtained from the same animal, pairing could not be consistently maintained across all experimental conditions due to predefined exclusion criteria (e.g., non-viable or damaged segments). Consequently, analyses were conducted using independent observations, with each vascular segment considered an experimental unit. This approach avoids selective pairing and maintains analytical consistency across datasets.

Sample size estimation was guided by effect sizes derived from preliminary vascular reactivity experiments and supported by power calculations performed using G*Power (version 3.1). Ethanol exposure produced a large effect size ($\eta^2 \approx 0.57$; Cohen's $f \approx 1.15$), supporting adequate power with small group sizes ($n = 3$–4; $\alpha = 0.05$; power = 0.80; four groups). In contrast, PVAT-related effects were of moderate magnitude ($\eta^2 \approx 0.11$; $f \approx 0.36$), requiring larger sample sizes ($n = 8$–10) for equivalent power. Accordingly, while the study was sufficiently powered to detect primary ethanol-induced effects, PVAT-dependent responses were interpreted in the context of their moderate effect sizes.

References

[1] Deaton RA, Bulut G, Serbulea V, Salamon A, Shankman LS, Nguyen AT, et al. A new autosomal Myh11-CreERT2 smooth muscle cell lineage tracing and gene knockout mouse model-brief report. *Arterioscler Thromb Vasc Biol*. 2023;43(2):203-211. doi:10.1161/ATVBAHA.122.318160

[2] Santos JD, Oliveira-Neto JT, Barros PR, Damasceno LEA, Lautherbach N, Assis AP, et al. Th17 cell-linked mechanisms mediate vascular dysfunction induced by testosterone in a mouse model of gender-affirming hormone therapy. *Am J Physiol Heart Circ Physiol*. 2022;323(2):H322-H335. doi:10.1152/ajpheart.00182.2022

[3] de Vicente LG, Muñoz VR, Pinto AP, Rovina RL, da Rocha AL, Marafon BB, et al. TLR4 deletion increases basal energy expenditure and attenuates heart apoptosis and ER stress but mitigates the training-induced cardiac function and performance improvement. *Life Sci*. 2021;285:119988. doi:10.1016/j.lfs.2021.119988

[4] Freitas A, Alves-Filho JC, Victoni T, Secher T, Lemos HP, Sônego F, et al. IL-17 receptor signaling is required to control polymicrobial sepsis. *J Immunol*. 2009;182(12):7846-7854. doi:10.4049/jimmunol.0803039

[5] Pérez MM, Martins LMS, Dias MS, Pereira CA, Leite JA, Gonçalves ECS, et al. Interleukin-17/interleukin-17 receptor axis elicits intestinal neutrophil migration, restrains gut dysbiosis and lipopolysaccharide translocation in high-fat diet-induced metabolic syndrome model. *Immunology*. 2019;156(4):339-355. doi:10.1111/imm.13028

[6] de Vicente LG, Pinto AP, Muñoz VR, Rovina RL, da Rocha AL, Gaspar RC, et al. Tlr4 participates in the responses of markers of apoptosis, inflammation, and ER stress to different acute exercise intensities in mice hearts. *Life Sci*. 2020;240:117107. doi:10.1016/j.lfs.2019.117107

[7] Silva CBP, Nascimento-Silva EA, Zaramela LS, da Costa BRB, Rodrigues VF, De Martinis BS, et al. Drinking pattern and sex modulate the impact of ethanol consumption on the mouse gut microbiome. *Physiol Genomics*. 2025;57(3):179-194. doi:10.1152/physiolgenomics.00031.2024
