# Supplementary material for: Gut–immune–PVAT axis is involved in ethanol-induced abdominal aortic dysfunction via IL-17RA, TLR4, and FPR1 signaling
Source: Gut Microbes. 2026 Sep 21;18(1):2734648. doi: 10.1080/19490976.2026.2734648 (PMC13596876; doi:10.1080/19490976.2026.2734648)
Supplement: Supplementary file 2 [file KGMI_A_2734648_SM7570.docx]

**SUPPLEMENTARY FIGURES**

**Supplementary Figure S1. Validation of smooth muscle cell-specific deletion of FPR1 in male mice.** (A) Representative agarose gel electrophoresis showing PCR-based genotyping for detection of the *Myh11-CreER^T2^* transgene and floxed *Fpr1* alleles. Band patterns confirm the presence of the Cre transgene and successful inheritance of floxed alleles. (B) PCR analysis confirming recombination of the floxed *Fpr1* allele following tamoxifen induction, using allele-specific primers. DNA ladder (Lad) and negative control (Neg) are shown. The presence of the recombined allele indicates efficient Cre-mediated excision in smooth muscle cells. (C) Representative immunofluorescence images of FPR1 expression in PVAT-intact thoracic aorta. FPR1 signal is markedly reduced in the medial layer of FPR1^fl/fl^/Myh11-CreER^T2^ mice compared to control mice, confirming efficient deletion of FPR1 in vascular smooth muscle cells (scale bar, 100 μm). Abbreviations: FPR1, formyl peptide receptor 1; Lad, DNA ladder; Neg, negative control; PCR, polymerase chain reaction; PVAT, perivascular adipose tissue; WT, wild-type.

**Supplementary Figure S2. Chronic plus binge ethanol exposure does not alter AA contractile responses in female WT mice.** Concentration–response curves to PE in AA without PVAT [PVAT (–)] from female WT mice (*n* = 7-8/group). Upper, representative curves. Lower, summary data showing E_max_, p*D*_2_, and AUC. Data were obtained from two independent experimental cohorts. Sample sizes vary due to technical limitations inherent to vascular reactivity assays. No significant differences were observed between groups (*p* > 0.05). Data are presented as mean ± SEM. Statistical analyses were performed using an unpaired Student’s *t*-test. Abbreviations: AA, abdominal aorta; AUC, area under the curve; E_max_, maximum response; p*D*_2_, -log EC_50_; PE, phenylephrine; PVAT, perivascular adipose tissue; WT, wild-type.

**Supplementary Figure S3. Flow cytometric gating strategy for immune cell identification.** (A, B) Representative gating strategy for neutrophils (A) and macrophage subsets (B) in abdominal perivascular adipose tissue (PVAT), including non-polarized (M0), classically activated (M1), and alternatively activated (M2) macrophages. (C) Representative gating strategy for T cell populations in cecal lymph nodes, including T helper 1 (Th1, Tbet), T helper 17 (Th17, Rorγt), and regulatory T (Treg, FoxP3) cells.

**Supplementary Figure S4. Intestinal inflammation and barrier integrity in male WT mice subjected to chronic, binge, or chronic plus binge ethanol exposure.** (A) Histopathological analysis of the colon (*n* = 5/group). Upper, representative H&E-stained sections (scale bar, 50 μm). Lower, histopathological inflammation score. (B–G) Colonic cytokine levels were determined by ELISA. Measurements for TNF-α, IL-6, IL-1β, and IL-10 (B–D, G) were obtained from the same set of samples (*n* = 6-7/group), while IL-17A and IL-22 (E–F) were measured in a separate common subset (*n* = 4-5/group). (H–N) RT-qPCR analysis of antimicrobial peptides – *Reg3a*, *Reg3b*, and *Reg3g* (*n* = 4-6/group) – and tight junction proteins – *occludin*, *claudin-2*, *claudin-3*, and *zonula occludens-*1 (*n* = 3-6/group) – in the colon. Gene expression was evaluated using the same samples. β-actin was used as the housekeeping gene. Data were obtained from one (A, E–F) or two (B–D, G–N) independent experimental cohorts. Sample sizes vary due to technical limitations inherent to each assay. Data are presented as mean ± SEM. Statistical analyses were performed using one-way ANOVA followed by Bonferroni’s post hoc test (A–D, F–N) or Kruskal-Wallis followed by Dunn’s post hoc test (E) (*p* < 0.05; exact *p* values are indicated). Abbreviations: H&E, hematoxylin and eosin; IL, interleukin; RT-qPCR, real-time quantitative PCR; TNF-α, tumor necrosis factor-alpha; WT, wild-type; ZO-1, zonula occludens-1.

**Supplementary Figure S5. Intestinal permeability, bacterial translocation, and systemic inflammation in male WT mice subjected to chronic, binge, or chronic plus binge ethanol exposure.** (A) Intestinal permeability assessed by serum FITC-dextran fluorescence (*n* = 6-9/group). (B) Bacterial translocation evaluated by CFU counts in whole blood (*n* = 4/group). (C–I) Systemic inflammation evaluated by plasma levels of inflammatory markers. Measurements for IL-1β, IL-6, TNF-α, and IL-10 (C–D, G, I) were obtained from the same set of samples (*n* = 7-8/group), IL-17A and IL-22 (E, H) were measured in a separate common subset (*n* = 5/group), and NO was measured in an additional sample set (*n* = 7-8/group) (F). Data were obtained from one (B), two (A, F) or three (C–E, G–I) independent experimental cohorts. Sample sizes vary due to technical limitations inherent to each assay. Data are presented as mean ± SEM. Statistical analyses were performed using one-way ANOVA followed by Bonferroni’s post hoc test (A–G, I) or Kruskal-Wallis followed by Dunn’s post hoc test (H) (*p* < 0.05; exact *p* values are indicated). Abbreviations: CFU, colony-forming unit; FITC, fluorescein isothiocyanate; IL, interleukin; NO, nitric oxide; TNF-α, tumor necrosis factor-alpha; WT, wild-type.

**Supplementary Figure S6. Chronic plus binge ethanol exposure reduces size and weight of spleen and kidney in male WT mice.** (A) Representative photographs of the abdominal cavity and gastrointestinal tract (one mouse per group). (B–C) Normalized colon (B) and cecum (C) lengths (*n* = 8/group). (D–E) Normalized heart (D) and liver (E) weights (*n* = 7-8/group). (F–G) Left, representative photographs of the spleen (F) and kidney (G) (three mice per group). Right, corresponding organ weights (*n* = 8/group). Data were obtained from three independent experimental cohorts. Lengths and weights were normalized to total body weight. Variations in sample sizes for heart weight (D) are due to the exclusion of statistical outliers identified by the ROUT method. Data are presented as mean ± SEM. Statistical analyses were performed using unpaired Student’s *t*-test (*p* < 0.05; exact *p* values are indicated). Abbreviations: WT, wild-type.

**Supplementary Figure S7. Chronic plus binge ethanol exposure increases aortic distensibility in male WT mice.** (A) Systolic blood pressure measured during treatment using the non-invasive tail-cuff method (*n* = 7-8/group). (B) Representative stress–strain curves of the AA obtained using a tissue puller system, along with summary data for AUC as an index of overall mechanical behavior. (*n* = 5/group). Data were obtained from one (A) or two (B) independent experimental cohorts. (C) Concentration–response curves to PE in suprarenal and infrarenal AA segments denuded of PVAT [AA-PVAT (–)] from WT control mice (*n* = 6-7/group). (D–E) Concentration–response curves to ACh (*n* = 8-9/group) (D) and SNP (*n* = 6-8/group) (E) in suprarenal AA-PVAT (–) segments from WT mice. Left, representative curves. Right, summary data showing E_max_ and AUC. Data for vascular reactivity assays were obtained from multiple experimental sessions (typically 2 animals per session) to achieve the final sample size, ensuring consistent experimental conditions across all groups. Sample sizes vary due to technical limitations inherent to each assay. Data are presented as mean ± SEM. Statistical analyses were performed using two-way ANOVA followed by Bonferroni’s post hoc test (A) or unpaired Student’s *t*-test (B–E) (*p* < 0.05; exact *p* values are indicated). Abbreviations: AA, abdominal aorta; ACh, acetylcholine; AUC, area under the curve; E_max_, maximum response; PE, phenylephrine; PVAT, perivascular adipose tissue; SNP, sodium nitroprusside; WT, wild-type.

**Supplementary Figure S8. Chronic plus binge ethanol exposure alters organ weights in male GF mice.** (A) Representative photographs of the gastrointestinal tract (one mouse per group). (B–C) Normalized colon (B) and cecum (C) lengths (*n* = 4-6/group). (D–G) Normalized organ weights: heart (D), spleen (E), kidney (F), and liver (G) (*n* = 4-6/group). Data were obtained from two independent experimental cohorts. Gastrointestinal lengths and organ weights were normalized to total body weight. Sample sizes vary due to the limited availability of GF mice. Data are presented as mean ± SEM. Statistical analyses were performed using unpaired Student’s *t*-test (A–F) or Mann-Whitney test (G) (*p* < 0.05; exact *p* values are indicated). Abbreviations: GF, germ-free.

**Supplementary Figure S9. Chronic plus binge ethanol exposure increases bacterial DNA content and shifts microbial composition in the abdominal PVAT.** (A) Plasma zonulin levels were measured by ELISA in WT, GF, and FPR1^-/-^ mice (*n* = 4-5/group), as an indirect marker of intestinal barrier integrity. (B–C) qPCR analysis of total bacterial 16S DNA levels (B) and the relative abundance of bacteria from Bacillota, Bacteroidota, Pseudomonadota, Actinomycetota, and Tenericutes phyla (C) within the abdominal PVAT from WT mice, representing bacterial translocation and altered local microbiota composition (*n* = 5-6/group). (C) For heatmap visualization, data were scaled relatively to the control group (set as white baseline), and deviations in ethanol-treated mice were represented by a color gradient. (D) Serum TMAO levels in WT mice were measured by ELISA (*n* = 9/group). Data were obtained from one (B**–**C) or three (A, D) independent experimental cohorts. Sample sizes vary due to technical limitations or limited sample availability. Data are presented as mean ± SEM. Statistical analyses were performed using two-way ANOVA followed by Bonferroni’s post hoc test (A, C) or unpaired Student’s *t*-test (B, D) (*p* < 0.05; exact *p* values are indicated). Abbreviations: FPR1^-/-^, FPR1^fl/fl^/Myh11-CreER^T2^; GF, germ-free; PVAT, perivascular adipose tissue; qPCR, quantitative PCR; TMAO, trimethylamine-N-oxide; WT, wild-type.

**Supplementary Figure S10. fMLP/FPR1 signaling modulates abdominal PVAT function under physiological conditions.** (A**–**B) Western blot analysis of FPR1 protein levels in abdominal PVAT lysates (30 µg/lane) from WT and GF mice. (A) Original immunoblot for FPR1 (38 kDa); (B) corresponding Ponceau-stained membrane (related to Fig. 2E). Samples were obtained from three independent treatment cohorts and analyzed in parallel to ensure experimental consistency. (C) Concentration–response curves to PE in AA segments with or without PVAT [PVAT (+) or PVAT (–)] from WT control mice in the presence of the FPR1 antagonist CsH or the agonist fMLP (10 µmol/L; *n* = 4-5/group). Upper, representative curves. Lower, summary data showing E_max_ and p*D*_2_. Data were obtained from four independent experimental cohorts. Sample sizes vary due to technical limitations inherent to vascular reactivity assays. Data are presented as mean ± SEM. Statistical analyses were performed using two-way ANOVA followed by uncorrected Fisher’s LSD test for multiple comparisons (*p* < 0.05; exact *p* values are indicated). Abbreviations: AA, abdominal aorta; C, control group; CsH, cyclosporin H; E_max_, maximum response; Et, chronic plus binge group; fMLP, N-formyl–methionyl–leucyl–phenylalanine; FPR1, formyl peptide receptor 1; GF, germ-free; p*D*_2_, -log EC_50_; PE, phenylephrine; PVAT, perivascular adipose tissue; WT, wild-type.

**Supplementary Figure S11. NOS inhibition increases contractile responses in AA and unmasks an anti-contractile PVAT phenotype.** (A–B) Concentration–response curves to PE in AA segments with or without PVAT [PVAT (+) or PVAT (–)] from WT control (A) or ethanol-treated (B) mice in the presence of vehicle (unincubated control) or the non-selective NOS inhibitor L-NAME (100 µmol/L; *n* = 5-7/group). Upper, representative curves. Lower, summary data showing E_max_ and p*D*_2_. Data were obtained from three to four independent experimental cohorts. Sample sizes vary due to technical limitations inherent to vascular reactivity assays. Data are presented as mean ± SEM. Statistical analyses were performed using two-way ANOVA followed by uncorrected Fisher’s LSD test for multiple comparisons (*p* < 0.05; exact *p* values are indicated). Abbreviations: AA, abdominal aorta; E_max_, maximum response; L-NAME, Nω-Nitro-L-arginine methyl ester; NOS, nitric oxide synthase enzyme; p*D*_2_, -log EC_50_; PE, phenylephrine; PVAT, perivascular adipose tissue; WT, wild-type.

**Supplementary Figure S12. Mitochondrial ROS contribute to AA hyporesponsiveness induced by chronic plus binge ethanol consumption in male WT mice.** (A–B) Concentration–response curves to PE in PVAT-denuded AA [AA-PVAT (–)] (*n* = 3-7/group) (A) or PVAT-intact AA [AA-PVAT (+)] (*n* = 4-5/group) (B) segments from control and ethanol-treated mice in the presence of vehicle or the mitochondria-targeted antioxidant MitoQ (100 nmol/L). Upper, representative curves. Lower, summary data showing E_max_ and p*D*_2_. Upper, representative curves. Lower, summary data showing E_max_ and p*D*_2_. Data were obtained from two to four independent experimental cohorts. Sample sizes vary due to technical limitations inherent to vascular reactivity assays. Data are presented as mean ± SEM. Statistical analyses were performed using two-way ANOVA followed by uncorrected Fisher’s LSD test for multiple comparisons (*p* < 0.05; exact *p* values are indicated). Abbreviations: AA, abdominal aorta; E_max_, maximum response; MitoQ, mitoquinone; p*D*_2_, -log EC_50_; PE, phenylephrine; PVAT, perivascular adipose tissue; WT, wild-type.
