# Supplementary material for: Gut–immune–PVAT axis is involved in ethanol-induced abdominal aortic dysfunction via IL-17RA, TLR4, and FPR1 signaling
Source: Gut Microbes. 2026 Sep 21;18(1):2734648. doi: 10.1080/19490976.2026.2734648 (PMC13596876; doi:10.1080/19490976.2026.2734648)
Supplement: Supplementary file 3 [file KGMI_A_2734648_SM7571.docx]

**SUPPLEMENTARY TABLES**

**Supplementary Table S1.** Primer sequences used for the genotyping assay to confirm smooth muscle cell-specific deletion of FPR1.

| Gene | Forward primer (5’-3’) | Reverse primer (5’-3’) | Company |
| --- | --- | --- | --- |
| 3’-loxP FPR1 | ATC CCT CTG TGA GAT GTA | ATG CAC ACA TGC AGG ACA TGC ACA G | Integrated DNA Technologies |
| 5’-loxP FPR1 | TCC AGT CTC AGA AAG TGA AGA GAT CCA | TCT GCC TGC CTC TTC CTC TCT TGT | Integrated DNA Technologies |
| Myh11 VSM Cre | TGA CCC CAT CTC TTC ACT CC | AGT CCC TCA CAT CCT CAG GTT | Integrated DNA Technologies |

**Supplementary Table S2.** Expected band sizes in the genotyping assay to confirm smooth muscle cell-specific deletion of FPR1.

| Strain | Mutant | Wild-type |
| --- | --- | --- |
| RKIP flox (EGE-ZWX-033-3’loxP-F/R) | 305 bp | 232 bp |
| RKIP flox (EGE-ZWX-033-5’loxP-F3/R3) | 382 bp | 310 bp |
| Myh11-CreERT2 | 287 bp | N/A |

Notes: bp: base pairs; N/A: not applicable.

**Supplementary Table S3.** Primer sequences used for qPCR analyses.

| Gene | Forward primer (5’-3’) | Reverse primer (5’-3’) | Company |
| --- | --- | --- | --- |
| *Reg3a* | GGCACCGAGCCCAATG | GGATTTCTCTCCCATGCAAAGT | Exxtend Biotecnologia |
| *Reg3b* | ATGCTGCTCTCCTGCCTGATG | CTAATGCGTGCGGAGGGTATATTC | Exxtend Biotecnologia |
| *REGIIIg* | TTCCTGTCCTCCATGATCAAAA | CATCCACCTCTGTTGGGTTCA | Exxtend Biotecnologia |
| *Occludin* | ATGTCCGGCCGATGCTCTC | TTTGGCTGCTCTTGGGTCTGTAT | Sigma-Aldrich |
| *Claudin-2* | TGCGACACACAGCACAGGCATCAC | TCAGGAACCAGCGGCGAGTAGAA | Exxtend Biotecnologia |
| *Claudin-3* | ACCAACTGCGTACAAGACGAG | CAGAGCCGCCAACAGGAAA | Sigma-Aldrich |
| *Zonula occludens-1* | TTCAAAGTCTGCAGAGACAATAGC | TCACATTGCTTAGTCCAGTTCC | Sigma-Aldrich |
| *Firmicutes* | ATGTGGTTTAATTCGAAGCA | AGCTGACGACAACCATGCAC | Sigma-Aldrich |
| *Bacteroidetes* | GTTTAATTCGATGATACGCGAG | TTAASCCGACACCTCACGG | Sigma-Aldrich |
| *Proteobacteria* | CATGACGTTACCCGCAGAAGAA | CTCTACGAGACTCAAGCTTGC | Sigma-Aldrich |
| *Actinobacteria* | TGTAGCGGTGGAATGCGC | AATTAAGCCACATGCTCCGCT | Sigma-Aldrich |
| *Tenericutes* | ATGTGTAGCGGTAAAATGCGTAA | CMTACTTGCGTACGTACTACT | Exxtend Biotecnologia |
| *Eubacteria universal* | ACTCCTACGGGAGGCAGCAGT | ATTACCGCGGCTGCTGGC | Exxtend Biotecnologia |
| *Beta-actin* | GGCCAACCGTGAAAAGATGA | CAGCCTGGATGGCTACGTACA | Exxtend Biotecnologia |

Notes: Nucleotide symbols: M = A or C; S = C or G.
